# Tissue-specific identification of multi-omics features for pan-cancer drug response prediction

**DOI:** 10.1016/j.isci.2022.104767

**Published:** 2022-07-19

**Authors:** Zhi Zhao, Shixiong Wang, Manuela Zucknick, Tero Aittokallio

**Affiliations:** 1 Institute for Cancer Research, Department of Cancer Genetics, Oslo University Hospital, Norway; 2 Centre for Biostatistics and Epidemiology (OCBE), Faculty of Medicine, University of Oslo, Norway; 3 Institute for Molecular Medicine Finland (FIMM), HiLIFE, University of Helsinki, Finland

**Keywords:** Drugs, Bioinformatics, Omics

## Abstract

Current statistical models for drug response prediction and biomarker identification fall short in leveraging the shared and unique information from various cancer tissues and multi-omics profiles. We developed mix-lasso model that introduces an additional sample group penalty term to capture tissue-specific effects of features on pan-cancer response prediction. The mix-lasso model takes into account both the similarity between drug responses (i.e., multi-task learning), and the heterogeneity between multi-omics data (multi-modal learning). When applied to large-scale pharmacogenomics dataset from Cancer Therapeutics Response Portal, mix-lasso enabled accurate drug response predictions and identification of tissue-specific predictive features in the presence of various degrees of missing data, drug-drug correlations, and high-dimensional and correlated genomic and molecular features that often hinder the use of statistical approaches in drug response modeling. Compared to tree lasso model, mix-lasso identified a smaller number of tissue-specific features, hence making the model more interpretable and stable for drug discovery applications.

## Highlights

• Pan-cancer cell lines provide a test bench for exploring gene-drug relationships
• Multi-omics data were integrated with pharmacological profiles for joint modeling
• Mix-lasso identifies tissue-specific biomarkers predictive of multi-drug responses
• Mix-lasso provides small number of stable features for drug discovery applications

## Graphical abstract

**Figure:**
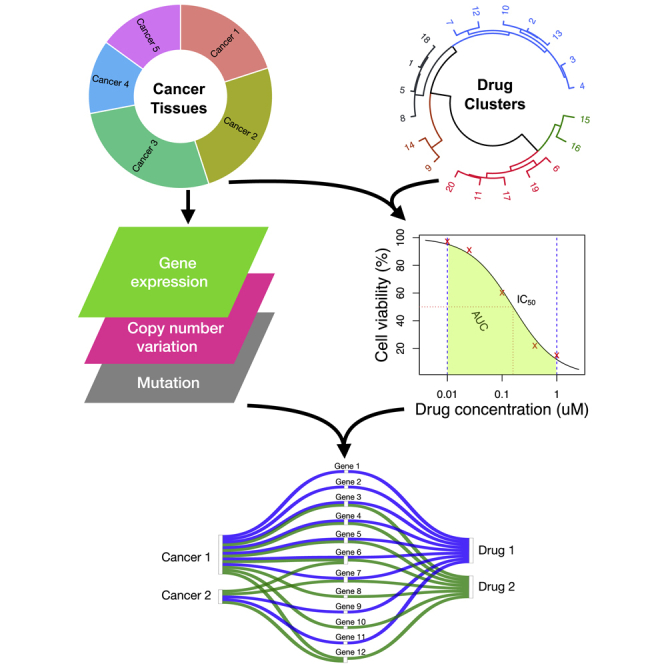

## Introduction

Targeted cancer therapies have been increasingly used during the past two decades for the treatment of certain cancer types that are driven by single oncogenic proteins (Druker et al., 2001; Tsimberidou et al., 2020); for example, HER2-positive breast cancer can be treated with HER2-targeted therapeutic agents such as trastuzumab (Vogel et al., 2002). However, our knowledge of such protein-therapy relationships is currently limited to only a few well-established links between specific cancer types and oncoprotein markers that can be used as companion diagnostics in the clinic. Personalized cancer medicine aims to target and use patient-specific genomic and molecular markers that drive the cancer or resistance development, and therefore determine the patient-specific responses to the available targeted treatments. However, due to the complexity of tumor biology and between-patient heterogeneity, targeted treatments may lead to differing or even opposite effects among patients with different cancer subtypes, yet having similar genetic or molecular backgrounds (Rowbotham et al., 2018; Gambardella et al., 2020).

Cancer tissue heterogeneity is critically important for modeling the potency and selectivity of targeted drugs across cancer types (Mannheimer et al., 2019; Lloyd et al., 2021). It has been shown that a drug inhibiting the same protein target may have drastically differing effects in different cancer tissues or cancer subtypes. For instance, inhibitors of the oncogene PI3K have shown to lead to highly varied effects (e.g., no response or extreme response) across different cancer types (Stewart et al., 2019). Therefore, a more systematic modeling of drug efficacy and identification of predictive markers beyond the target proteins requires simultaneous analysis of pharmacogenomic data from multiple cancer types or subtypes. In particular, there is a need for predictive models that can accurately estimate the tissue-specific effects on drug response profiles through integrating still rather limited sample sizes of heterogeneous cancer (sub)types, with the aim of predicting multi-drug responses by simultaneously taking into account the most relevant genomic and molecular features in a pan-cancer setting.

There are a number of publicly available data resources for large-scale pharmacogenomic screens, which include hundreds of cancer cell lines from multiple cancer types, treated with hundreds of drugs and characterized at baseline (before treatment) with multiple omics data such as gene expression, copy number variation, and point mutations (Barretina et al., 2012; Seashore-Ludlow et al., 2015). In these screens, drug response is summarized based on a dose-response curve to quantitatively score how effectively the drug inhibits cell growth, for example, using the half-maximal inhibitory concentration (IC_50_) or the area under the drug dose-response curve (AUC). Even though the cell line models cannot capture all the variability seen in patient tumors, these large-scale data resources provide great opportunities for estimating or even predicting of drug efficacy in a pan-cancer setting and for the development of novel statistical models for this task. However, the heterogeneous nature of the pharmacogenomic data poses challenges for predictive drug response modeling. These challenges include multivariate responses involving drug-drug similarities and frequent missing values, heterogeneous and partly unknown cancer tissues and subtypes (e.g., hidden sub-groups), high-dimensional genomic features with gene-gene correlations, and heterogeneous multi-omics profiles.

A number of statistical and machine learning models have been developed in the past years for predicting drug responses (see e.g., Ballester et al. (2022); Sharifi-Noghabi et al. (2021); Adam et al. (2020)). These models are often designed for making accurate predictions, either within a single tissue (Costello et al., 2014) or using a tissue-agnostic approach (Barretina et al., 2012), and most of the models cannot deal with missing data and other technical variability present in the high-throughput studies. Furthermore, while emphasizing accurate predictions, many of the models lack effective feature selection options, making such black-box models less practical for biological studies or clinical applications. Previously, Kim and Xing (2012) proposed tree-guided group lasso (tree lasso) for multi-response regression that leverage a hierarchical structure over multiple response variables to select relevant covariates from high-dimensional features. However, tree lasso cannot deal with heterogeneity between multiple sample groups. Huang et al. (2020) developed Tissue-guided LASSO (TG-lasso) for integrating cancer tissue of origin with genomic profiles. However, the TG-lasso pipeline repeats the analysis in each tissue type, rather than jointly modeling multiple cancer types.

To address these limitations, we developed a tissue-specific lasso model that takes advantage of our IPF-tree-lasso (Tree-guided group lasso with Integrative Penalty Factors) to capture drug-drug similarities and deal with heterogeneous high-dimensional multi-omics data (Zhao and Zucknick, 2020). For short, we call our approach mix-lasso, where the mix refers to both mixed models, a mix of multi-omics data sources, and a mix of multiple cancer types. In comparison to the existing models, the newly developed mix-lasso considers the predictive contributions of heterogeneous cancer types by borrowing the methodology from varying-coefficient mixed models (Hoover et al., 1998). To leverage pan-cancer information of the same genomic or molecular features, the tissue-specific effects are taken into account by grouping effects using the elastic net penalty (Zou and Hastie, 2005), which enables robust selection of sparse sets of multi-omics features (or markers) most predictive of drug responses across cancer types. In contrast to many other statistical models, mix-lasso can effectively deal with unmeasured drug responses, which are missing-at-random in the high-throughput screens, to make full use of the drug response profiles of each cancer type. The optimization applies the smoothing proximal gradient (SPG) method, similarly to the tree lasso (Kim and Xing, 2012), which is used as a reference comparison model in our study.

## Results

The Cancer Therapeutics Response Portal (CTRP) v2 is a database of large-scale cancer cell line drug screens (Seashore-Ludlow et al., 2015; Basu et al., 2013). In CTRP v2, the responses of 481 drugs are profiled across 860 cell lines from 24 primary tumor types. The genomic and molecular information of the cell lines originates from the Cancer Cell Line Encyclopedia (CCLE, Barretina et al. (2012)), including genome-wide measurements of mRNA expression, DNA copy number variation, and DNA point mutations. In the response modeling, we made use of the log2 intensity values for the genome-wide mRNA expression data (Affymetrix Human Genome U133 Plus 2.0 arrays), log2 ratio values for the genome-wide copy number variation (Affymetrix SNP Array 6.0), and binary values for the gene point mutations of selected cancer gene loci measured using mass spectrometric genotyping (OncoMap platform). We used the following criteria to preselect subsets of the drugs, cell lines, and genomic features.

• Every cancer type must have at least 15 cell lines for tissue-specific modeling, which was earlier considered large enough sample size for comparison of pan-cancer and tissue-specific prediction models (Lloyd et al., 2021).
• A part of the selected drugs must have a completely measured response sub-matrix across cell lines, which was used for a direct comparison between mix-lasso, that allows for missing responses, and tree lasso, that does not allow missing responses.
• Among gene expression (GEX) features, we selected the most variable features, without any missing data, such that ≥ 50% of the cumulative variance is included, similar to our previous work (Zhao and Zucknick, 2020).
• Among copy number variation (CNV) features, we selected the most variable features, without any missing data, such that ≥ 50% of the cumulative variance is included, similar to our previous work (Zhao and Zucknick, 2020).
• Mutation (MUT) features include all genes that harbor deleterious single point mutations from CCLE and have pathogenic mutation scores according to COSMIC (Catalog of Somatic Mutations In Cancer) (Tate et al., 2018), without any missing data, by following Barretina et al. (2012) and Garnett et al. (2012).

To construct the hierarchical structure of drug-drug similarity for mix-lasso’s IPF-tree penalty (see Equation 4 in STAR Methods), we preselected a complete response dataset from the total 481 drugs by excluding about half of the drugs with missing values. Since mix-lasso can deal with missing data, we included as many cell lines as possible. Finally, the above preselection criteria led to ∼ 200 drugs (Note that this is not an exact number, because we randomly split the cell lines into learning and validation datasets 10 times, where in every run the learning dataset forms a different complete drug response matrix with around 200 cell lines and around 200 drugs. Our analysis results focus on 147 common drugs across the 10 repeats.) and 473 cell lines from 20 cancer types as our pharmacogenomic profiling dataset. We used higher resolution histologic subtypes of primary lung tumor, due to strong heterogeneity within the primary lung cancers. The multi-omics profiling data included a total of 2069 GEX features, 8127 CNV features, and 175 MUT features preselected as input data for modeling.

**Figure 1:**
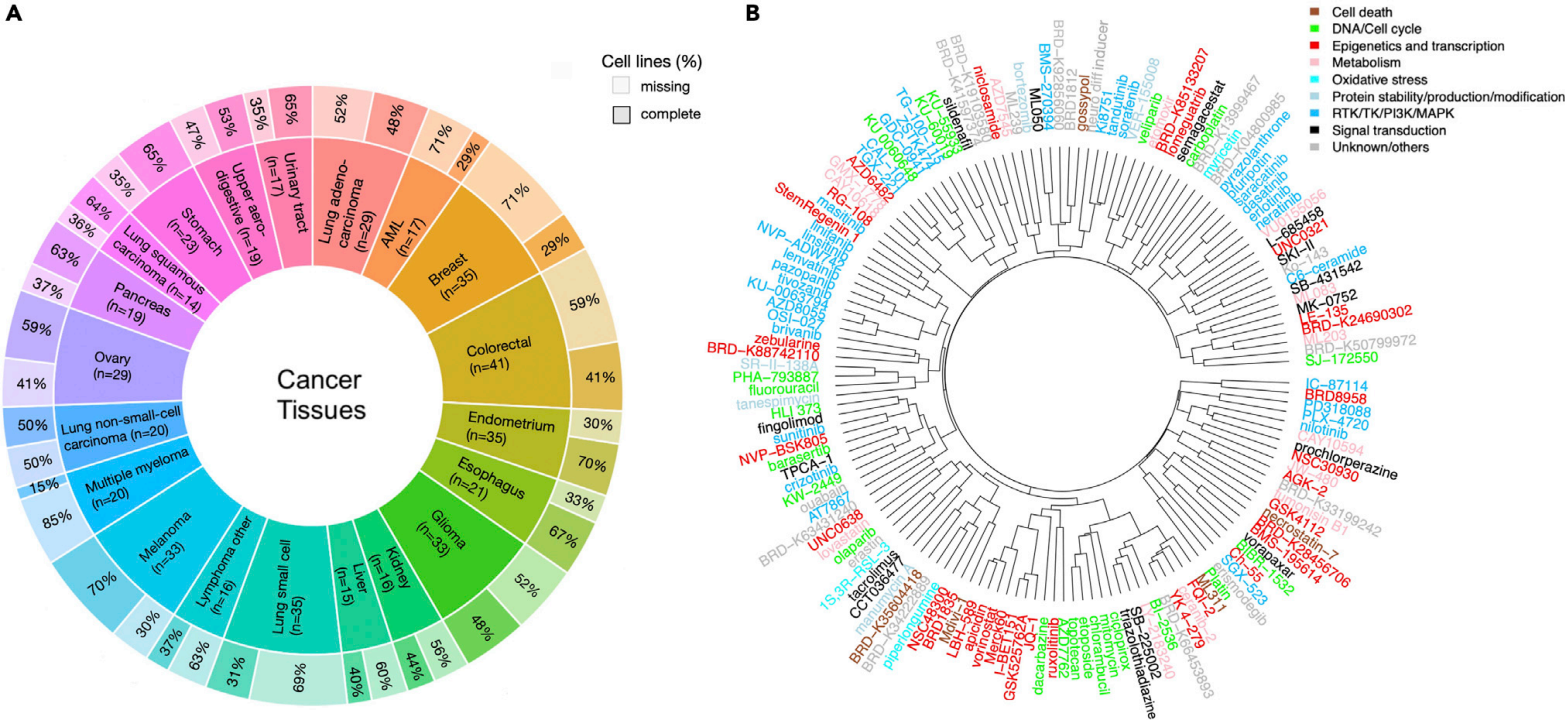
Distribution of the 20 cancer types and a hierarchical clustering of the drugs from CTRP (A) The number of cell lines from each cancer type/tissue is shown in parentheses. The label “complete” denotes the portion of cell lines with complete drug response data, and “missing” the portion with some missing values in the drug response data. (B) Clustering of the drug responses was carried out using hierarchical agglomerative complete-linkage clustering with Pearson correlation similarity measure for drug responses (AUC profiles over the cell lines). The 147 drugs shown are the common drugs shared across the repeated analyses used in the feature selection analyses below (among 10 repetitions). The colors indicate the mode of action (MoA) classes of the drugs (see Figure S1.1 for an alternative clustering of the drugs based on their structural similarity).

Figure 1A shows the distribution of the 473 cell lines across 20 cancer types (Note that lung squamous cell carcinoma has 14 cell lines, which is fewer than the threshold of 15 in our criteria, because a few cell lines without complete genomic data were removed.), with median proportion of missing values across cancer types 51% (range 30% ∼ 85%). Less than half of the cell lines have complete drug response data, making the prediction task relatively challenging. To quantify the drug response outcome, we used the area under the drug dose-response curve (AUC) according to recent guidelines Sharifi-Noghabi et al. (2021). Figure 1B shows that the clustering of the drug responses based on the similarity of the AUC profiles across the cell lines only partly corresponds to the mode of action (MoA) classes of the drugs.

### Prediction accuracy and feature selection performance

For the model evaluation, the cell lines of each cancer tissue type were split into 75% for training data and 25% for validation data. We randomly split the data into the two parts 10 times for investigating the stability of the selected features and for evaluating the average prediction performance for drug responses. A genomic or molecular feature was determined to be selected by a model if its estimated regression coefficient was nonzero at least 2 (or 5) times out of the 10 repeats. Note that we counted multiple times whether the same gene was selected as a predictor for multiple drugs, since there are both common and drug-specific predictive features among the three omics data sources (i.e., gene expression, copy number variation, and point mutations).

When using all three omics data sources, the copy number variation features did not contribute markedly to the overall drug response predictions with mix-lasso (Figure S1.2). After removing the copy number variation data, mix-lasso improved its overall prediction accuracy across the 147 drugs, w.r.t. Root Mean Squared Error (RMSE) and had similar Pearson and Spearman correlations, whereas tree lasso remained at similar level of overall prediction accuracy (Table S1). Figures S1.3 and S1.4 show the overlapping GEX and MUT features, selected by mix-lasso, when modeling either three or two omics data (i.e. removing copy number variation features); the relatively small portion of unique features identified only when modeling all the three omics data (highlighted in red) indicates that modeling of the two omics data captured most of the predictive signal from the three omics data, and suggests that the additional features selected from the GEX and MUT data compensated for the effect of the missing CNV features in the mix-lasso model. Therefore, we only use the two omics data sources, i.e., gene expression and point mutations, in the following analyses.

**Figure 2:**
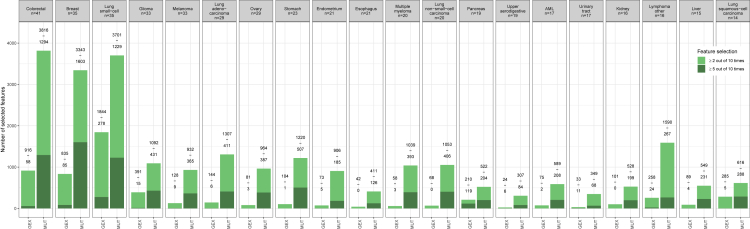
Feature selection with mix-lasso across the $m_{\text{drugs}}=147$ common drugs w.r.t. the two omics data sources (GEX and MUT) and 20 cancer types (the columns) The total numbers of features for individual data sources were $m_{\text{drugs}}\times p_{\text{GEX}}\approx 3.0\times 10^{5}$ and $m_{\text{drugs}}\times p_{\text{MUT}}\approx 2.6\times 10^{4}$. The two values above each bar show the numbers of features selected (i.e. nonzero coefficients; note that each feature can be counted multiple times if it was selected as a predictor for multiple drugs across the 20 cancer types). The light green (or dark green) bar shows the numbers of features selected when one of the model coefficients was nonzero at least 2 (or at least 5) out of 10 repetitions. The cancer types are ordered by sample size from colorectal to lung squamous cell carcinoma.

Figure 2 shows the feature selection performance across the 20 cancer types by the mix-lasso model. Interestingly, the point mutations were more commonly selected for overall drug response prediction by mix-lasso, even if the total number of potential GEX features was more than 10-fold higher than that of MUT features. When comparing the two frequency criteria of feature selection (i.e., ≥2 and ≥5 out of 10 times), mutation features were also more stably selected than the gene expression features, as measured by the Lance-Williams distance (16.9 for GEX vs. 9.8 for MUT) (The Lance-Williams distance measures a distance between two vectors of the numbers of selected features for one omics data source over the cancer tissue types based on criteria ≥2 and ≥5 out of 10 times, i.e., two vectors $x_{i}$ and $y_{i}$ (i=1,⋯,n) have distance $\sum_{i=1}^{n}\frac{\left|x_{i}-y_{i}\right|}{\left|x_{i}\right|+\left|y_{i}\right|}$. The smaller the Lance-Williams distance, the more similar are the two vectors.). This indicates that the mix-lasso selects a rather stable set of mutation features for drug response prediction, which are likely to provide more practical biomarker panels, compared to gene expression markers that are more challenging to use as companion diagnostics in clinical practice.

**Figure 3:**
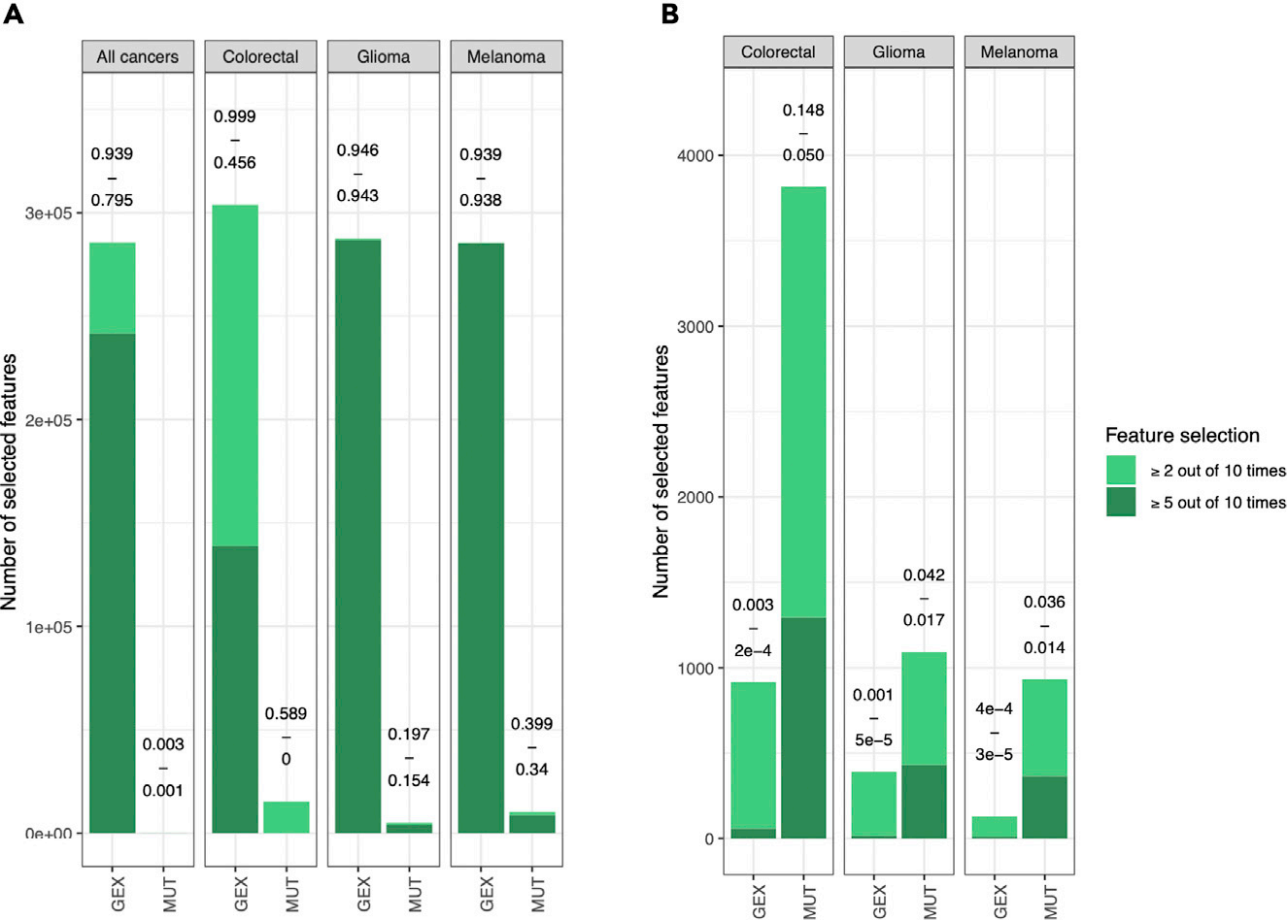
Feature selection with tree lasso and mix-lasso (A) Feature selection with tree lasso across the $m_{\text{drugs}}=147$ common drugs, either by modeling all cancer tissue types jointly (left panel) or by separately modeling each cancer type where possible (only cancer types with enough samples with complete drug response data could be used for tree lasso modeling). The two values above each bar show the estimated model sparsity (i.e., $\frac{\text{number}\;\text{of}\;\text{selected}\;\text{features}}{\text{number}\;\text{of}\;\text{all}\;\text{potential}\;\text{features}}$). The light green (or dark green) bar shows the numbers of features selected when one of the model coefficients was nonzero at least 2 (or at least 5) out of 10 repetitions. (B) Feature selection with mix-lasso for the same tissue types and $m_{\text{drugs}}=147$ common drugs. Note: the y-axis scale is different between the two panels, while the percentages above the bars are more comparable between the two models.

In comparison with mix-lasso, tree lasso resulted in much denser models (i.e., a more complex model with more selected features) for drug response prediction, which may become less practical in translational applications, where sparse models with fewer selected features are preferred. When modeling the complete dataset with all cancer types, tree lasso selected 95% and 79% of the GEX features based on the criteria “≥2 out of 10 times” and “≥5 out of 10 times”, respectively (first panel Figure 3A). Similarly, dense models were estimated with tree lasso when separately modeling individual cancer types, which was possible for the three largest cancer types (colorectal, glioma, and melanoma; Figure 3A), which had sufficient sample size (n>15 in complete drug response data) for tree lasso modeling. Notably, tree lasso selects 94% of all gene expression features for glioma and melanoma with both of the selection criteria. In contrast, mix-lasso results in reasonably sparse models for gene expression and mutation features (Figure 3B). Taken together, these results demonstrate that the mix-lasso model is able to identify sparse and robust subsets of tissue-specific genomic and molecular features for multi-drug response prediction in a pan-cancer setting.

### Prediction accuracy across cancer tissues and MoA classes

To investigate in more detail the prediction performance of mix-lasso and tree lasso using all cancer types, we evaluated the rank correlation between measured AUCs and predicted AUCs for each drug and each cancer type using the two models (Figure S1.5). Interestingly, mix-lasso and tree lasso showed rather complementary prediction accuracy across the cancer types; for instance, mix-lasso predicted accurately more drug responses in colorectal (Figure S1.5a) and ovarian cancer (Figure S1.5g), whereas tree lasso made more accurate response predictions for a number of drugs in stomach (Figure S1.5h) and lung squamous cell carcinoma cancer (Figure S1.5s). Moreover, the accuracy of neither of the methods was dependent on whether predicting targeted or non-targeted drugs, rather the methods showed again rather complementary prediction accuracies across the cancer types and drug classes (Figure S1.6). These results suggest that the two models compensate each other in the sense that when tree lasso performs poorly, the mix-lasso makes accurate predictions, and vice versa (Figure S1.7).

**Figure 4:**
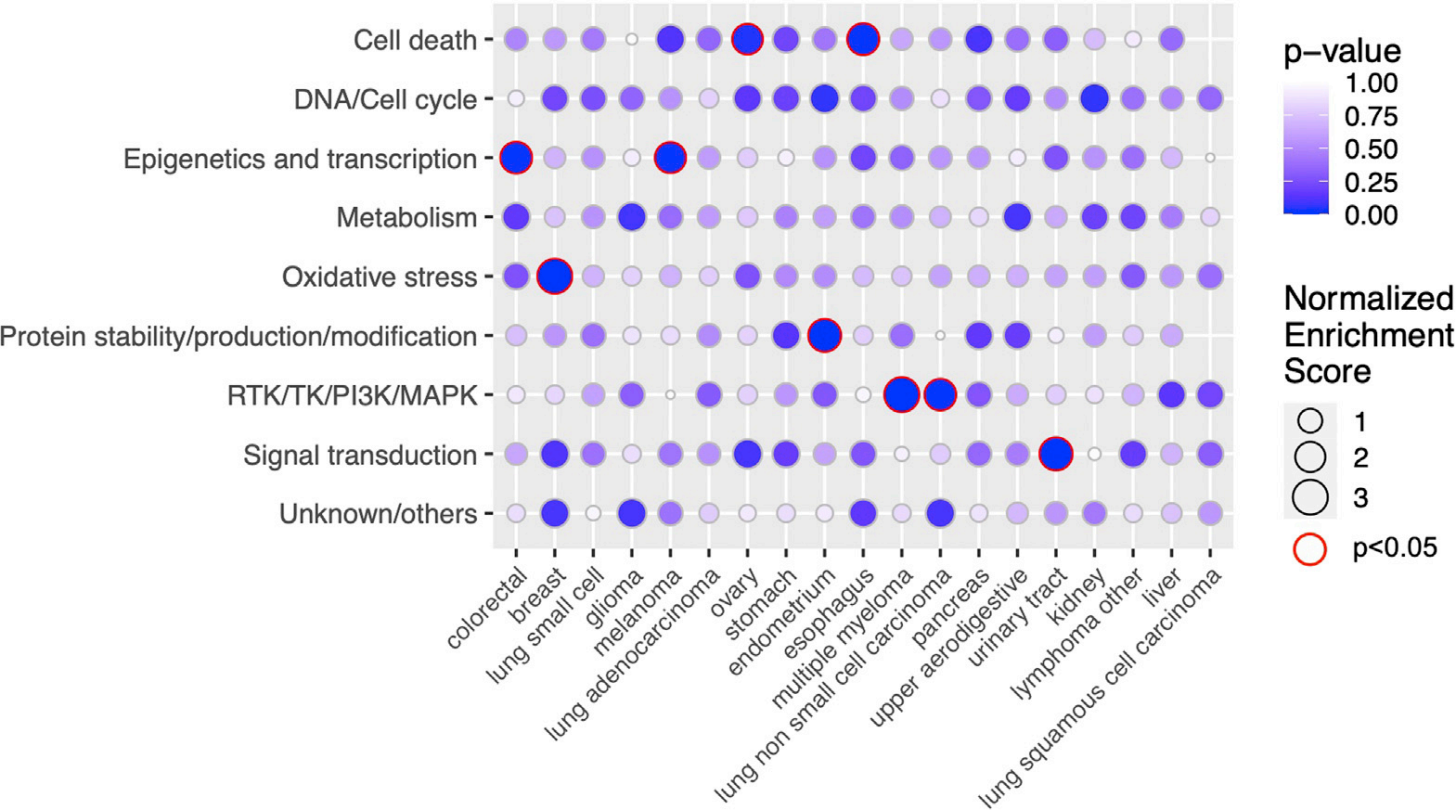
Drug set enrichment analysis across individual cancer tissue types and 9 drug MoA classes, where the 147 drugs were ranked by prediction accuracy of mix-lasso (Spearman correlation) The size of a circle corresponds to a normalized enrichment score (Kolmogorov-Smirnov statistic). No circles mean missing p-value because of not enough variation in the predicted Spearman’s ρ. Due to the relatively small number of drugs in the enrichment analysis, the false discovery rate was not controlled.

**Figure 5:**
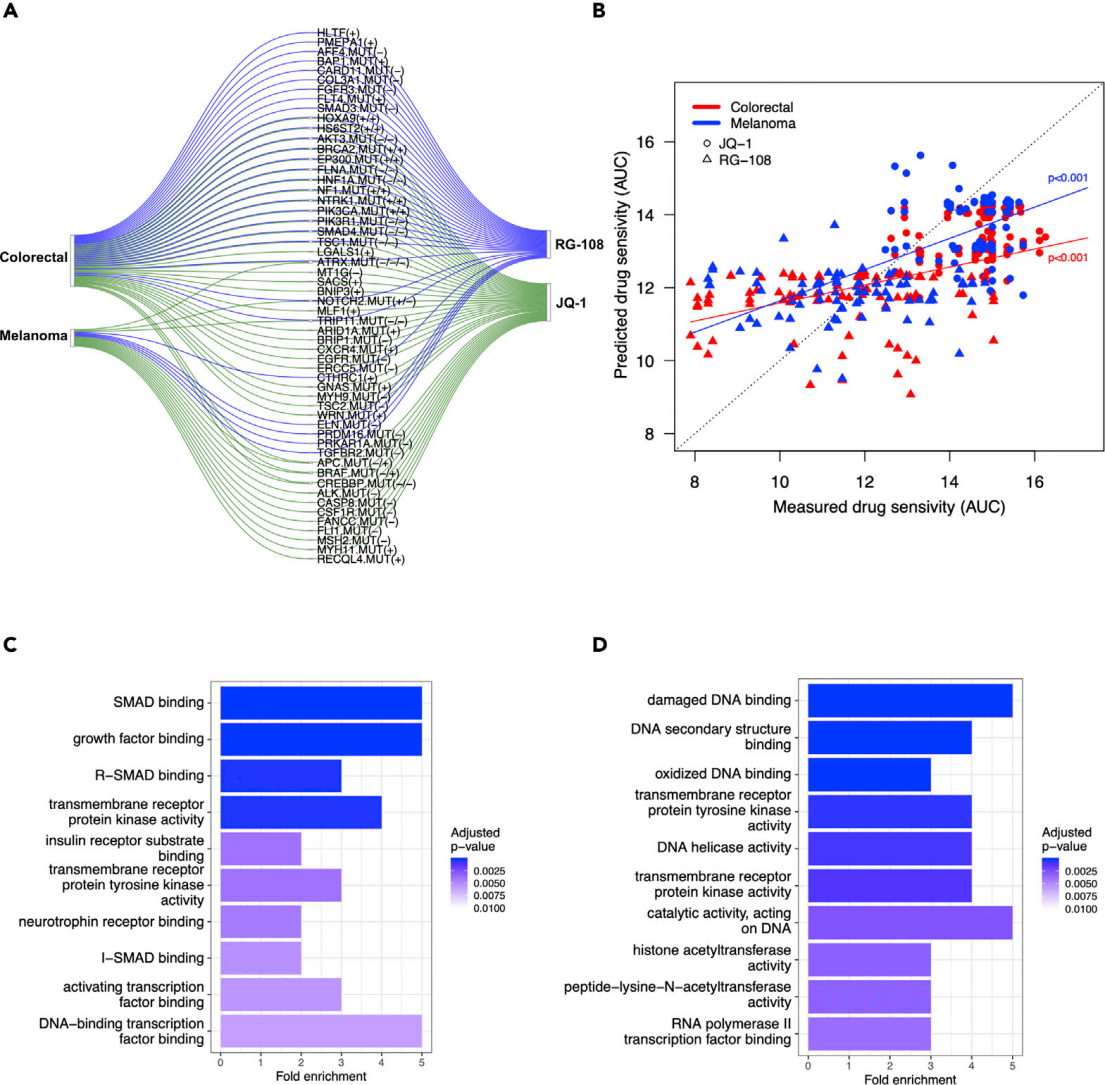
Set of genes predictive of drug responses and their enrichment analysis (A) Example drugs RG-108 and JQ-1 and their identified genomic (MUT) and molecular features (GEX) linked to the two selected cancer types by mix-lasso. The selected genes were based on feature selection criteria “≥2 out of 10 times”. Gene names with “.MUT” indicate MUT features and the rest are GEX features. “+” or “-” indicates positive or negative effect. The multiple signs correspond to distinct gene-cancer-drug response relationships (i.e., the number of connections in the sankey diagram). Note that the negative effect of “NOTCH2.MUT” corresponds to drug JQ-1 in melanoma, and the negative effect of “BRAF.MUT” corresponds to drug JQ-1 in colorectal cancer. (B) Relationships between the measured drug response (area under the drug dose-response curve, AUC) and the predicted drug response using mix-lasso model in the validation data. The dotted diagonal line indicates perfect prediction of the drug response. The two colored lines indicate regression lines for the two selected cancer types, respectively, and the p-values show the significance of the regressions. Enrichment of GO molecular functions among the mix-lasso-selected genes predictive of responses to (C) RG-108 and (D) JQ-1. p-values in panels (C) and (D) were adjusted for multiple testing by controlling the false discovery rate with the Benjamini & Hochberg method.

To investigate potential differences in the prediction accuracies across mode of action (MoA) drug classes, we grouped the drugs into 9 broad MoA classes based on their targets or inhibition mechanisms. We then applied drug set enrichment analysis (Napolitano et al., 2016) to investigate whether the mix-lasso can predict well certain classes of drugs for specific cancer tissue types (Figure 4). For example, we observed that epigenetic and transcription modulating drugs can be predicted accurately for colorectal cancer and melanoma (p<0.05, Figure 4). Previous studies have also suggested that compounds that act epigenetically or modulate transcription may provide potential therapies for these cancer types (Patnaik and Anupriya, 2019; Jung et al., 2020; Strub et al., 2020; Giunta et al., 2021; Garcia-Gomez et al., 2021). As a specific example, we chose RG-108 and JQ-1 from this drug class and investigated their selected predictive features in the two cancer types (Figure 5). Even though the target proteins of RG-108 and JQ-1 were not selected by the mix-lasso model, the selected features listed in Figure 5A are connected to the drugs’ target activity via Gene Ontology (GO) set enrichment analysis (Figures 5C and 5D).

For instance, it is known that the TGF-β-SMAD pathway (Wotton, 2012; Papageorgis et al., 2010; Bai and Xi, 2018) and transcription factor binding (Figure 5C) are closely related to the RG-108 target DNA methyltransferase. Similarly, the targets of JQ-1, i.e., the BET family of bromodomain proteins, are active players in transcription and epigenetics, and they can promote cancer cell proliferation and survival. In Figure 5D, enriched molecular functions, such as DNA binding and transcription factor binding, are closely related to the function of BET proteins as direct transcriptional regulators, and molecular functions of receptor tyrosine kinases and tyrosine kinases also reflect the role of BET proteins in mediating the transcription from various signals that promote cell proliferation. Furthermore, JQ-1-related proteins are also enriched in chromatin-associated cellular components, e.g., chromosome and telomeric regions (Figure S1.8), which also relate to the function of BET proteins in transcription and epigenetics. These results indicate that non-target proteins or other proteins in the target pathways predict the responses of RG-108 and JQ-1 in a tissue-specific manner.

Another example from the drug set enrichment analysis is the enrichment of RTK/TK/PI3IK/MAPK drug class in multiple myeloma (MM) and lung non-small-cell carcinoma (Figure 4). We therefore investigated IC-87114, PLX-4720, and TG-100-115 from this drug class in these two different types of cancers (Figure S1.9). IC-87114 and TG-100-115 are specific PI3Kδ/γ inhibitors, and previous studies have shown the effectiveness of targeting different PI3K isoforms in MM (Piddock et al., 2017; Sahin et al., 2014; Glauer et al., 2013; Ikeda et al., 2010) (Figure S1.9a). Even though the hierarchical clustering did not cluster the two PI3K inhibitors based on drug response (Figure 1B), their Spearman correlation is significant (Spearman’s ρ=0.111, p=0.021). The genomic features that are linked to the responses of the two drugs are also biologically relevant (Figure S1.9a); for example, EGFR is the upstream factor of PI3Ks, and the effectiveness of combined CDK4/6 (targets of CDKN2A) and PI3K inhibition has been shown in other cancer models (O’Brien et al., 2020; Bonelli et al., 2017). The GO molecular functions of identified genes for drugs IC-87114 and TG-100-115 both include phosphatidylinositol 3′-kinase activity (Figures S3.9b and S3.8d). PLX-4720 is a BRAF inhibitor, and some of the identified features (e.g., MET, KRAS, and NF1) are either closely related to the signal initiation and transduction of the MAPK pathway, in which BRAF is involved in, or downstream effector molecules (e.g., MAX and MYC) of the RTK/MAPK pathway (Figure S1.9d).

## Discussion

In this study, we introduced the mix-lasso model to jointly analyze multi-omics data from *in vitro* pharmacogenomic screens in cell lines. Such integrative models are needed in the preclinical anticancer drug discovery process to evaluate the relationship between somatic variation and preclinical treatment responses, which can further guide future screening of phenotypic effects of anticancer compounds in preclinical model systems (Ballester et al., 2022). Large-scale pharmacogenomic screens have been carried out to date in hundreds of cancer cell line panels to provide insights into drug efficacy and potential molecular and genomic determinants of context-specific drug response through the omics profiling among the various cancer tissue types (Barretina et al., 2012; Seashore-Ludlow et al., 2015; Haverty et al., 2016). When analyzed using suitable statistical approaches, these rich data resources are expected to help finding biomarkers predictive of preclinical drug responses that can be followed-up in future studies.

However, there has been lack of effective approaches for tissue-specific statistical modeling and sparse feature selection in the pharmacogenomic data involving heterogeneous pan-cancer information and multiple omics data sources (Ali and Aittokallio, 2019; Adam et al., 2020). To that end, we proposed the mix-lasso model and demonstrated how it enables successful applications to datasets composed of mix of multiple cancer tissue types and multiple omics measurements. Mix-lasso provides a useful and timely modeling approach, since increasing number of cancer cell lines and associated phramacogenomic data for many tissue types are becoming available from the ongoing and emerging screening efforts, and we expect that the same integrated modeling approach can effectively integrate the current and future multi-omics data, with the aim to identify potential tissue-specific multi-omics features for further preclinical or clinical studies.

There exist only a few studies that have addressed the challenges posed by tissue-specific modeling based on pan-cancer multi-omics pharmacogenomic data (Mannheimer et al., 2019; Huang et al., 2020; Naulaerts et al., 2020; Lloyd et al., 2021). However, these previous studies have analyzed each cancer tissue type dataset separately, which makes it difficult to leverage the pan-cancer information and to distinguish between common and tissue-specific molecular and genomic features across multiple cancer types. Although useful statistical methodologies have also been developed in the past for complex structured data with integrated high-dimensional genomic information (see Ickstadt et al., 2018; Reel et al., 2021), most of these models lack options for capturing certain important structures in the complex data, including heterogeneity across sample groups. Many of the existing models cannot either deal with missing data, which is inherent to large-scale screens.

In many real-world applications, there exist heterogeneous sample groups that have opposite effects of the same features on treatment responses (e.g., in sub-groups of cancer patients). For instance, Figure 5A shows that BRAF mutant gene has negative effect on the response of JQ-1 in colorectal cancer, which has been reported earlier Nakamura et al. (2017), while having a positive effect on the response of a structurally similar compound I-BET151 in melanoma (Figures 5B and S1.10), which has been shown in a previous study Gallagher et al. (2014). In such cases, the proposed mix-lasso model was shown to improve both the identification of relevant features and prediction of treatment response in comparison with a reference tree lasso model in the simulation studies (Figure S2.1). If all the samples are relatively homogeneous, then there is no need for group/tissue-specific feature selection, and the standard tree lasso or IPF-tree-lasso model is sufficient.

Our results in the real-world CTRP data demonstrated that mix-lasso provides more interpretable feature selection results in terms of much fewer number of selected genes, with different features selected for different cancer tissue types and more stable feature selection results, compared to tree lasso, which selected almost all the gene expression features and had less stable mutation feature selection (Figure 3). In particular, a small number of stably selected point mutations can be expected to lead to practical companion diagnostics in translational applications, compared to gene expression levels that are often more difficult to use in clinical practice. Although mix-lasso resulted in highly sparse models with only a few selected genes, it still predicted accurately the responses of specific classes of drugs for many cancer tissue types (Figures 4 and S1.5a–S1.5s). This is partly because the selected genes were shown to be related to the target pathways or other MoA mechanisms of the predicted drugs.

### Limitations of the study

Our selection criteria for the CTRP dataset might lead to biased results, because some cancer types do not have many cell lines, which may limit statistical power; missing drug responses might be not truly missing-at-random; and some of the filtered gene expression and mutation features with low variance might turn out to be important for drug response modeling. Similar to many other drug response prediction models (Ali and Aittokallio, 2019; Adam et al., 2020; Koras et al., 2020), mix-lasso was not able to make effective use of copy number variation information to predict drug responses. This might be because neighboring copy number variation features share strong correlations, and since copy number variation is often anticorrelated with point mutations (Iorio et al., 2016), making it difficult to distinguish their predictive contributions. A possible extension of mix-lasso is to employ a fused-lasso penalty for copy number variation features (Cheng et al., 2018). A related limitation of the current mix-lasso model is its limited capability to capture the exact relationships between the predictive features across different omics data sources. This could be addressed in the future studies by further employing group-lasso penalties corresponding to correlated features across different omics data sources, for example, grouping effects of GEX, CNV, and MUT of the same gene by penalizing $\|\beta_{\text{GEX}}+\beta_{\text{CNV}}+\beta_{\text{MUT}}{\|}_{\ell_{2}}$. Any prior knowledge of correlated features within one omics data source can also be addressed in the same way.

In addition to gene expression, mutations, and copy number variation, the mix-lasso model is also applicable to a broader set of omics data sources. Since drug response and resistance is known to be determined by complex genetic and epigenetic factors, it will be important to include other types of multi-omics input data, including protein modifications (Ali et al., 2018), gene isoforms (Safikhani et al., 2017), metabolite profiling (Daemen et al., 2015), and even microbiome data, once such data become available for multiple tissue types. Another way to improve drug response prediction and to search for response-predictive biomarkers would be to use protein-target and pathway information already in the feature selection process (Koras et al., 2020; Ben-Hamo et al., 2020). While patient tumors are beyond the scope of multi-drug testing, mix-lasso should be easily applicable to drug profiling in patient cells (*ex vivo*, Letai et al. (2022)) and animal models (*in vivo*, Nguyen et al. (2021)).

## STAR★Methods

### Key resources table

**Table:** 

REAGENT or RESOURCE	SOURCE	IDENTIFIER
**Deposited data**		

Pharmacological profiling,i.e. the area under thedrug-dose response curve	The Cancer TherapeuticsResponse Portal (CTRP)	https://ocg.cancer.gov/programs/ctd2/data-portal
Multi-omics data of CTRP,i.e. gene expression (GEX), copy number variation (CNV),point mutation (MUT)	PharmacoDB	From R/Bioconductor package**PharmacoGx** directly
**Software and algorithms**		

R3.6.0	This study	https://www.r-project.org
mix-lasso	This study	https://github.com/zhizuio/mixlasso

### Resource availability

#### Lead contact

Further information and requests for resources and reagents should be directed to and will be fulfilled by the lead contact, Tero Aittokallio (t.a.aittokallio@medisin.uio.no).

#### Materials availability

This study did not generate new unique reagents.

### Method details

#### Prediction problem and model formulation

Let us suppose drug responses are profiled in *n* cell lines for *m* drugs, hence forming a n×m drug response matrix Y. The *n* cell lines originate from *T* cancer tissue types, or in general *T* sample groups (e.g., patient samples), and the *t*-th tissue type has $n_{t}$ samples, $\sum_{t=1}^{T}n_{t}=n$. We further suppose that high-dimensional genomic and molecular features originate from *S* omics data sources (e.g., gene expression, copy number variation and point mutations), in total $p=\sum_{s=1}^{S}p_{s}$. The *t*-th cancer tissue type has a multi-omics predictor matrix

$$\left[X_{\left(t\right)1},\cdots ,X_{\left(t\right)s},\cdots ,X_{\left(t\right)S}\right]=X_{\left(t\right)}=\left\{x_{\left(t\right)ij}\right\},\;\left(i=1,\cdots ,n_{t};j=1,\cdots ,p_{s}\right)$$

where $X_{\left(t\right)s}$ is a $n_{t}\times p_{s}$ matrix representing the *s*-th omics data source corresponding to the *t*-th tissue type. The full omics data $X=\left[X_{\left(1\right)}\vdots \ldots \vdots X_{\left(T\right)}\right]$ is constructed by stacking by rows.

To predict the multi-drug responses Y using the multi-omics profiling data X, it is necessary to take into account (i) drug-drug similarities (i.e. correlations between the response variables) that may mutually support the prediction of correlated drugs, and (ii) gene-gene correlations and heterogeneity between the multi-omics data sources that include jointly or separately predictive features. We previously proposed a structured penalized regression model, IPF-tree-lasso (Zhao and Zucknick, 2020), which captures these two aspects, but not the heterogeneous contribution of multiple cancer tissue types to drug response modeling. IPF-tree-lasso builds on multivariate linear regression, and it minimizes the sum of squares of residuals (Frobenius norm) of differences between predicted and measured responses, penalized by a IPF-tree-structure penalty term:

(Equation 1) $$\frac{1}{2mn}{\left\|Y-1_{n}\beta_{0}^{⊤}-XB\right\|}_{F}^{2}+pen_{\text{IPF}-\text{tree}}\left(B\right),$$

where the penalty term $pen_{\text{IPF}-\text{tree}}\left(B\right)$ uses integrative penalty factors to penalize different omics data sources differently (Boulesteix et al., 2017) and uses a tree-structure $\ell_{1}/\ell_{2}$-penalty (Kim and Xing, 2012) to take into account a hierarchical structure of correlations between Y columns, which encourages the model to identify similar sets of genomic and molecular features for drugs with similar responses.

#### Mix-lasso model

Here, we introduce varying coefficients into the IPF-tree-lasso model, which makes it possible to estimate tissue-specific feature effects in a pan-cancer setting. For the *t*-th cancer tissue dataset $Y_{\left(t\right)}$ and $X_{\left(t\right)}$, we estimate the tissue-specific feature effect matrix $B_{\left(t\right)}$ through a linear model

$$Y_{\left(t\right)}=1_{n_{t}}\beta_{\left(t\right)0}^{⊤}+X_{\left(t\right)}B_{\left(t\right)}+E_{\left(t\right)},$$

where $\beta_{\left(t\right)0}$ denotes the intercept vector and $E_{\left(t\right)}=\left(\varepsilon_{\left(t\right)1},\cdots ,\varepsilon_{\left(t\right)m}\right)$ is a noise matrix with each column $\varepsilon_{\left(t\right)•}∼N\left(0,\sigma_{\varepsilon }^{2}I_{n_{t}}\right)$. However, since the drug response profiles from the same cancer tissue are often correlated, a random effect $u_{t}∼N\left(0,\sigma_{u}^{2}\right)$ is added to take into account the correlations between drug responses. The joint model of all cancer tissue types data becomes

(Equation 2) $$\left[\begin{array}{l} Y_{\left(1\right)}\\ \vdots \\ Y_{\left(T\right)} \end{array}\right]=\left[\begin{array}{l} 1_{n_{1}}\beta_{\left(1\right)0}\\ \vdots \\ 1_{n_{T}}\beta_{\left(T\right)0} \end{array}\right]+\left[\begin{array}{l} X_{\left(1\right)}B_{\left(1\right)}\\ \vdots \\ X_{\left(T\right)}B_{\left(T\right)} \end{array}\right]+Zu1_{m}^{⊤}+\left[\begin{array}{l} E_{\left(1\right)}\\ \vdots \\ E_{\left(T\right)} \end{array}\right]$$

where Z is a n×T dummy variable of the cancer tissue types, $u={\left(u_{1},\cdots ,u_{T}\right)}^{⊤}$. We also assume the random effects $u_{t}$ and noise terms $E_{\left(t\right)}$ (t=1,⋯,T) are mutually independent.

Instead of directly minimizing the (penalized) sum of squared residuals, similarly to the IPF-tree-lasso or tree-lasso, we need to maximize the penalized log likelihood function to account for the random effects in the tissue-specific IPF-tree-lasso model. The negative log likelihood of the *t*-th tissue corresponding to the *k*-th drug response variable $y_{\left(t\right)k}$ is

(Equation 3) $$-\ell \left(y_{\left(t\right)k};\beta_{\left(t\right)0},\beta_{\left(t\right)k},\sigma_{u}^{2},\sigma_{\varepsilon }^{2}\right)=\frac{n_{t}}{2}\log\left(2\pi \right)+\frac{1}{2}\log\left|V\left(t\right)\right|-\frac{1}{2}{\left(y_{\left(t\right)k}-1_{n_{t}}\beta_{\left(t\right)0}-X_{\left(t\right)}\beta_{\left(t\right)k}\right)}^{⊤}V_{\left(t\right)}^{-1}\left(y_{\left(t\right)k}-1_{n_{t}}\beta_{\left(t\right)0}-X_{\left(t\right)}\beta_{\left(t\right)k}\right),$$

where the covariance matrix $V_{\left(t\right)}$ is $n_{t}\times n_{t}$ dimensional with diagonals $\sigma_{u}^{2}+\sigma_{\varepsilon }^{2}$ and off-diagonals $\sigma_{u}^{2}$. The variance of the random effect $\sigma_{u}^{2}$ is a nuisance parameter, since we focus on prediction of drug responses and feature selection (i.e. estimation of feature effects), rather than correlation within a cancer tissue type. The variance $\sigma_{u}^{2}$ is not straightforward to estimate because of often limited sample sizes of each cancer tissue in practice, and computational challenges associated with simultaneous estimation of the high-dimensional feature effects. To simplify the optimization problem, we use a proxy $\tilde{V}=I_{n}+ZMZ^{⊤}$ for $\text{diag}\left\{V_{\left(1\right)},\cdots ,V_{\left(T\right)}\right\}$, where $M=\left(\text{log}n\right)I_{T}$. Fan and Li (2012) proved in linear mixed effects models that the proxy matrix ensures the model selection consistency, i.e., weak oracle property of coefficient estimators in the sense of Lv and Fan (2009). A slightly different proxy with $M=\frac{2}{3T}I_{T}$ was proposed by Bradic et al. (2020), which does not result in model selection consistency, but has a slightly higher power for the fixed effects in simulations.

For the purpose of drug response prediction, the random effect $u_{t}$ (t=1,⋯,T) can be predicted by the maximum a posteriori principle which is essentially its conditional mean given data and model parameters. We need this estimator for predicting a differing effect for each cancer type, since the average effect across all cancer types is zero. Similar to Schelldorder et al. (2011), we define

$${\tilde{u}}_{t}=\underset{u_{t}}{\text{argmin}}\;f\left(u_{t}|Y_{1},\cdots ,Y_{\left(t\right)},\beta_{\left(t\right)0},B,\sigma_{u}^{2}\right)\;=\underset{u_{t}}{\text{argmin}}\;\frac{f\left(Y_{\left(t\right)}|u_{t},\beta_{\left(t\right)0},B_{\left(t\right)},\sigma_{u}^{2}\right)f\left(u_{t}\right)}{f\left(Y_{\left(t\right)}|\beta_{\left(t\right)0},B_{\left(t\right)},\sigma_{u}^{2}\right)}\;=\underset{u_{t}}{\text{argmin}}\left\{\sum_{k=1}^{m}\frac{1}{\sigma_{\varepsilon }^{2}}\|y_{\left(t\right)k}-1_{n_{t}}\beta_{\left(t\right)0}-X_{\left(t\right)}\beta_{\left(t\right)k}-1_{n_{t}}u_{t}{\|}^{2}+u_{t}^{2}/\sigma_{u}^{2}\right\}\;={\left(m1_{n_{t}}^{⊤}1_{n_{t}}+\sigma_{\varepsilon }^{2}/\sigma_{u}^{2}\right)}^{-1}1_{n_{t}}^{⊤}\sum_{k=1}^{m}\left(y_{\left(t\right)k}-1_{n_{t}}\beta_{\left(t\right)0}-X_{\left(t\right)}\beta_{\left(t\right)k}\right),$$

where *f* is the density of the corresponding Gaussian distributed variable. The $\sigma_{\epsilon }^{2}/\sigma_{u}^{2}$ can be obtained by $M^{-1}$ in the proxy matrix of Fan and Li (2012), and ${\hat{\beta }}_{\left(t\right)0}$ and ${\hat{\beta }}_{\left(t\right)k}$ are estimated by the Smoothing proximal gradient (SPG) method proposed in tree lasso (Kim and Xing, 2012). From this $u_{t}$ is predicted by

$${\hat{u}}_{t}={\left(m1_{n_{t}}^{⊤}1_{n_{t}}+{\left(\text{log}n\right)}^{-1}\right)}^{-1}1_{n_{t}}^{⊤}\sum_{k=1}^{m}\left(y_{\left(t\right)k}-1_{n_{t}}{\hat{\beta }}_{\left(t\right)0}-X_{\left(t\right)}{\hat{\beta }}_{\left(t\right)k}\right).$$

The model (Equation 2) estimates multiple tissue-specific effects of each genomic and molecular feature on prediction of a particular drug response. The model also allows for grouping effects of multiple effects originating from the same feature, for example, one gene may have similar effects in multiple cancer types. For the *j*-th gene corresponding to the *k*-th drug, one needs to estimate the regression coefficients $\beta_{\left(1:T\right)jk}={\left(\beta_{\left(t\right)jk},\cdots ,\beta_{\left(T\right)jk}\right)}^{⊤}$. A sparse group lasso penalty (Simon et al., 2013) is used for the grouping effect of $\beta_{\left(1:T\right)jk}$, i.e., $\left(1-\alpha \right)\gamma \sqrt{T}{\left\|\beta_{\left(1:T\right)jk}\right\|}_{\ell_{2}}+\alpha \gamma {\left\|\beta_{\left(1:T\right)jk}\right\|}_{\ell_{1}}$, where γ>0, α∈[0,1] and $\|\beta_{\left(1:T\right)jk}{\|}_{\ell_{q}}={\left(\sum_{t=1}^{T}{\left|\beta_{\left(t\right)jk}\right|}^{q}\right)}^{1/q}$ ($q\in N^{+}$). For $\ell_{q}$-norm of a matrix, ${\left\|B\right\|}_{\ell_{q}}={\left(\sum_{k=1}^{m}\sum_{j=1}^{p}{\left|\beta_{jk}\right|}^{q}\right)}^{1/q}$.

Finally, the mix-lasso model has the objective function

(Equation 4) $$-\sum_{t=1}^{T}\sum_{k=1}^{m}\ell \left(\beta_{\left(t\right)0},\beta_{\left(t\right)k},\sigma_{u}^{2},\sigma_{\varepsilon }^{2}\right)+\sum_{t=1}^{T}\sum_{s=1}^{S}\sum_{j_{s}=1}^{p_{s}}\lambda_{s}\sum_{\nu \in V_{\text{int}}}\omega_{v}{\left\|\beta_{\left(t\right)j_{s}}^{G_{v}}\right\|}_{\ell_{2}}+\sum_{t=1}^{T}\sum_{s=1}^{S}\sum_{j_{s}=1}^{p_{s}}\lambda_{s}\sum_{\nu \in V_{\text{leaf}}}\omega_{v}{\left\|\beta_{\left(t\right)j_{s}}^{G_{v}}\right\|}_{\ell_{2}}+\left(1-\alpha \right)\gamma \sum_{k=1}^{m}\sum_{j=1}^{p}\sqrt{T}{\left\|\beta_{\left(1:T\right)jk}\right\|}_{\ell_{2}}+\alpha \gamma {\left\|B\right\|}_{\ell_{1}}.$$

The 1st term is the sum of negative log-likelihoods in (Equation 3) over multiple sample groups. The 2nd and 3rd terms are the IPF-tree penalty, in which a tree of drug responses with a set of vertices *V* and groups $\left\{G_{v}:v\in V\right\}$, *V* consists of internal nodes $V_{\text{int}}$ and leaf nodes $V_{\text{leaf}}$, and $\beta_{\left(t\right)j_{s}}^{G_{v}}$ are coefficients corresponding to predictors $X_{\left(t\right)j_{s}}$ in the *s*-th data source across response group $G_{v}$ (see Zhao and Zucknick (2020) for details). If $\lambda_{s}=\lambda$ (s=1,⋯,S), then the 3rd and 5th terms together simplify to $\left(\lambda +\alpha \gamma \right){\left\|B\right\|}_{\ell_{1}}$, since $\omega_{v}=1$ when $\nu \in V_{\text{leaf}}$ and the heights of the dendrogram are normalized. To apply the SPG method for model optimization, we smooth the penalty term $\gamma \sum_{k=1}^{m}\sum_{j=1}^{p}\sqrt{T}{\left\|\beta_{\left(1:T\right)jk}\right\|}_{\ell_{2}}$ and the IPF-tree-lasso penalty terms involving internal nodes.

#### Optimization of mix-lasso

Multiple data sources of predictors can be easily transformed to an equivalent problem of one data source, see Zhao and Zucknick (2020). We here only provide details of the optimization of mix-lasso with one data source of predictors. Mix-lasso with one data source of predictors has the following objective function

$$-\sum_{t=1}^{T}\sum_{k=1}^{m}\ell \left(\beta_{\left(t\right)0},\beta_{\left(t\right)k},\sigma_{u}^{2},\sigma_{\varepsilon }^{2}\right)+\lambda \left\{\sum_{t=1}^{T}\sum_{j=}^{p}\sum_{\nu \in V_{\text{int}}}{\left\|\omega_{\nu }\beta_{\left(t\right)j}^{G_{\nu }}\right\|}_{\ell_{2}}+\sum_{t=1}^{T}\sum_{j=1}^{p}\sum_{\nu \in V_{\text{leaf}}}{\left\|\omega_{\nu }\beta_{\left(t\right)j}^{G_{\nu }}\right\|}_{\ell_{2}}\right\}+\left(1-\alpha \right)\gamma \sum_{k=1}^{m}\sum_{j=1}^{p}\sqrt{T}{\left\|\beta_{\left(1:T\right)jk}\right\|}_{\ell_{2}}+\alpha \gamma {\left\|B\right\|}_{\ell_{1}},$$

where

$$-\ell \left(\beta_{\left(t\right)0},\beta_{\left(t\right)k},\sigma_{u}^{2},\sigma_{\varepsilon }^{2}\right)=\frac{n_{t}}{2}\log\left(2\pi \right)+\frac{1}{2}\log\left|{\text{V}}_{\left(\text{t}\right)}\right|-\frac{1}{2}{\left(y_{\left(t\right)k}-1_{n_{t}}\beta_{\left(t\right)0}-X_{\left(t\right)}\beta_{\left(t\right)k}\right)}^{⊤}V_{\left(t\right)}^{-1}\left(y_{\left(t\right)k}-1_{n_{t}}\beta_{\left(t\right)0}-X_{\left(t\right)}\beta_{\left(t\right)k}\right).$$

For the covariance matrix, we can use a plug-in proxy matrix $\tilde{V}$ suggested by Fan and Li (2012) or Bradic et al. (2020). Then we modify the smoothing proximal gradient (SPG) method proposed by Kim and Xing (2012). Combining the tree lasso penalty involving internal nodes and the grouped-tissue penalty, we have

$$\text{Ω}\left(B\right):=\lambda \sum_{t=1}^{T}\sum_{j=1}^{p}\sum_{\nu \in V_{\text{int}}}\omega_{\nu }{\left\|\beta_{\left(t\right)j}^{G_{\nu }}\right\|}_{\ell_{2}}+\left(1-\alpha \right)\gamma \sum_{k=1}^{m}\sum_{j=1}^{p}\sqrt{T}{\left\|\beta_{\left(1:T\right)jk}\right\|}_{\ell_{2}}=\lambda \sum_{t=1}^{T}\sum_{j=1}^{p}\sum_{\nu \in V_{\text{int}}}\omega_{\nu }\underset{{\left\|\alpha_{j}^{G_{\nu }}\right\|}_{\ell_{2}}\leq 1}{\text{max}}{\left(\alpha_{j}^{G_{\nu }}\right)}^{⊤}\beta_{\left(t\right)j}^{G_{\nu }}+\left(1-\alpha \right)\gamma \sum_{k=1}^{m}\sum_{\nu \in K_{\left(t\right)}}\underset{{\left\|\alpha_{k}^{\text{∗}K_{\nu }}\right\|}_{\ell_{2}}\leq 1}{\text{max}}{\left(\alpha_{j}^{\text{∗}K_{\nu }}\right)}^{⊤}\beta_{k}^{K_{\nu }}=\sum_{t=1}^{T}\underset{A\in Q}{\text{max}}\langle CB_{\left(t\right)}^{⊤},A\rangle +\underset{A^{\text{∗}}\in Q^{\text{∗}}}{\text{max}}\langle C^{\text{∗}}B,A^{\text{∗}}\rangle ,$$

where *C*, A, $C^{\text{∗}}$ and $A^{\text{∗}}$ are

$$\begin{array}{l} C_{\left(\nu ,i\right)}^{l}=\left\{ \begin{array}{ll} \omega_{\nu } & \text{if}\;l\;\in \;G_{V_{\text{int}}}\\ 0 & \text{otherwise} \end{array}\right.,C_{\left(\nu ,i\right)}^{\text{∗}l}=\left\{ \begin{array}{ll} 1 & \text{if}\;l\;\in \;K_{K_{\left(t\right)}}\\ 0 & \text{otherwise} \end{array}\right.,\\ A=\left[\begin{array}{lll} \alpha_{1}^{G_{1}} & \ldots & \alpha_{p}^{G_{1}}\\ \vdots & \ddots & \vdots \\ \alpha_{1}^{G_{\left|V_{\text{int}}\right|}} & \ldots & \alpha_{p}^{G_{\left|V_{\text{int}}\right|}} \end{array}\right],A^{\text{∗}}=\left[\begin{array}{lll} \alpha_{1}^{\text{∗}K_{1}} & \ldots & \alpha_{m}^{\text{∗}K_{1}}\\ \vdots & \ddots & \vdots \\ \alpha_{1}^{\text{∗}K_{1}} & \ldots & \alpha_{m}^{\text{∗}K_{p}} \end{array}\right]. \end{array}$$

The smooth approximation to the nonsmooth penalty Ω(B) is

$$f_{\mu }\left(B\right)=\sum_{t=1}^{T}\left(\underset{A\in Q}{\text{max}}\langle CB_{\left(t\right)}^{⊤},A\rangle -\mu d\left(A\right)\right)+\underset{A^{\text{∗}}\in Q^{\text{∗}}}{\text{max}}\langle C^{\text{∗}}B,A^{\text{∗}}\rangle -\mu d\left(A^{\text{∗}}\right),$$

and its gradient is

$$\nabla f_{\mu }\left(B_{\left(t\right)}\right)=A_{\left(t\right)1}^{⊤}C+C^{\text{∗}⊤}A_{2},$$

where $A_{\left(t\right)1}={\left(\alpha_{j}^{G_{\nu }}\right)}^{⋆}=S\left(\frac{\lambda \omega_{\nu }\beta_{\left(t\right)j}^{G_{\nu }}}{\mu }\right)$, $A_{2}={\left(\alpha_{k}^{\text{∗}K_{\nu }}\right)}^{⋆}=S\left(\frac{\left(1-\alpha \right)\gamma \sqrt{T}\beta_{k}^{K_{\nu }}}{\mu }\right)$, S(⋅) is the shrinkage operator. Note that the same $A_{2}$ is applied to different sample groups, which induces similar gradients for effects of different sample groups.

Let the smoothing (penalized) likelihood be

$$h\left(B\right)=-\sum_{t=1}^{T}\sum_{k=1}^{m}\ell \left(\beta_{\left(t\right)0},\beta_{\left(t\right)k},\sigma_{u}^{2},\sigma_{\varepsilon }^{2}\right)+f_{\mu }\left(B\right).$$

Its gradient w.r.t. the intercept and coefficients of the *t*-th sample group is

$$\nabla h\left(\beta_{\left(t\right)0}^{⊤},B_{\left(t\right)}\right)=X_{\left(t\right)}^{\text{∗}⊤}V_{\left(t\right)}^{-1}X_{\left(t\right)}^{\text{∗}}\left[\begin{array}{l} \beta_{0,t}^{⊤}\\ B_{\left(t\right)} \end{array}\right]-X_{\left(t\right)}^{\text{∗}⊤}V_{\left(t\right)}^{-1}Y_{\left(t\right)}+\left[\begin{array}{lll} 0 & 0 & 0\\ 0 & A_{1,t}^{⊤} & A_{2}^{⊤} \end{array}\right]\left[\begin{array}{l} 0\\ \lambda I_{p}C\\ \left(1-\alpha \right)\gamma I_{p}C^{\text{∗}} \end{array}\right],$$

which is Lipschitz continuous with Lipschitz constant

$$L_{\left(t\right)}=\lambda_{\text{max}}\left(X_{\left(t\right)}^{\text{∗}⊤}V_{\left(t\right)}^{-1}X_{\left(t\right)}^{\text{∗}}\right)+\frac{1}{\mu }{\left\|\left[\begin{array}{l} 0\\ \lambda I_{p}C\\ \left(1-\alpha \right)\gamma \sqrt{T}I_{p} \end{array}\right]\right\|}^{2}.$$

Let $B_{\left(t\right)}=W_{\left(t\right)}^{\left(b\right)}-\frac{1}{L_{\left(t\right)}}\nabla h\left(W_{\left(t\right)}^{\left(b\right)}\right)$. By second-order Taylor approximation,

$$h\left(B_{\left(t\right)}\right)\approx h\left(W_{\left(t\right)}^{\left(b\right)}\right)+\langle B_{\left(t\right)}-W_{\left(t\right)}^{\left(b\right)},\nabla h\left(W_{\left(t\right)}^{\left(b\right)}\right)\rangle +\frac{L_{\left(t\right)}}{2}{\left\|B_{\left(t\right)}-W_{\left(t\right)}^{\left(b\right)}\right\|}_{\ell_{2}}^{2}.$$

According to the proximal gradient method and calculating the order of subgradient, we can obtain a closed-form solution of (b+1)-th iterated $B_{\left(t\right)}^{\left(b+1\right)}$

$$\begin{array}{l} \beta_{\left(t\right)0}^{⊤}=\omega_{0,t}^{⊤\left(b\right)}-\frac{1}{L_{\left(t\right)}}\nabla h\left(\omega_{0,t}^{⊤\left(b\right)}\right),\\ \beta_{\left(t\right)jk}=\text{sign}\left(w_{\left(t\right)jk}\right)\text{max}\left(0,\left|w_{\left(t\right)jk}\right|-\frac{\lambda \omega_{\nu \left(k,t\right)}}{L_{\left(t\right)}}\right), \end{array}$$

where $w_{\left(t\right)jk}$’s (j=1,⋯,p) are the elements of $W_{\left(t\right)}^{\left(b\right)}-\frac{1}{L_{\left(t\right)}}\nabla h\left(W_{\left(t\right)}^{\left(b\right)}\right)$.

#### Missing drug response data

In practice, some of the cancer cell lines may not be treated with all the drugs, or some of the drug assays may have failed for technical reasons or been removed in the quality control phase, which results in missing data in the drug response matrix Y. If we can assume that the data are missing-at-random, we can make use of all the available data for multi-drug response modeling, including the cell lines and drug responses where some values are missing. We use a projection operator Π(⋅) to project the missing entries to zeros similarly to Li et al. (2019). In practice, calculating the residuals between responses Y and linear predictors $1_{n}{\hat{\beta }}_{0}^{⊤}+X\hat{B}$ in the penalized likelihood (Equation 1) becomes

$$\text{Π}\left(Y-1_{n}{\hat{\beta }}_{0}^{⊤}-X\hat{B}\right),$$

which only takes into account non-missing drug response data and ignores missing entries. If $Y_{ik}$ is missing, ${\left\{\text{Π}\left(\cdot \right)\right\}}_{ik}=0$; otherwise ${\left\{\text{Π}\left(\cdot \right)\right\}}_{ik}={\left\{Y-1_{n}\beta_{0}^{⊤}-XB\right\}}_{ik}$. We note that this technique is used when calculating a Frobenius norm or quadratic form of $Y-1_{n}\beta_{0}^{⊤}-XB$ when optimizing the objective function (Equation 4) of mix-lasso.

#### Benchmarking simulation study

To evaluate the performance of the proposed mix-lasso and to compare it against a reference method, tree lasso, we simulated *m* response variables, *n* samples from *T* sample groups and *p* potential features. A comparison between tree lasso, IPF-tree-lasso and other lasso-type methods for multi-omics data was carried out in Zhao and Zucknick (2020), so we only use tree lasso as a reference method in this study. The penalty parameters of mix-lasso and tree lasso were optimized using 3-fold cross-validation among the *n* simulated samples, which would in real-world applications correspond to cancer cell lines or patient-derived primary samples, for example.

The simulation data of the *t*-th group (t=1,⋯,T) are generated by

$$\begin{array}{l} x_{\left(t\right)i}∼N\left(0_{p\times 1},{\text{Σ}}_{X}\right),\\ Y_{\left(t\right)}∼N\left(X_{\left(t\right)}B_{\left(t\right)},I_{m}⊗V_{\left(t\right)}\right), \end{array}$$

where ${\text{Σ}}_{X}$ is designed in the same way as in Zhao and Zucknick (2020) to simulate correlated features, and $B_{\left(t\right)}$ is a sparse structured matrix to generate responses with tree-structure relationships; see Kim and Xing (2012) or Zhao and Zucknick (2020) for more details.

In the simulated settings, we set m=120, n=300, T=10, p=1000, ${\text{Σ}}_{X}$ with diagonals 1 and off-diagonals of 10 diagonal blocks 0.4, $V_{\left(t\right)}$ with diagonals 1 and off-diagonals 0.5, and $B_{\left(t\right)}$ has the same tree structure as the design in Zhao and Zucknick (2020), including 1800 out of mp=120000 nonzero coefficients for each sample group. In each setting, we assume 5% randomly missing drug responses. We further consider various practical settings for other parameters to mimic large-scale pharmacogenomic screens:

• **Scenario 1**: nonzero coefficients of $B_{\left(t\right)}$ (t=1,⋯,T) are **0.5.**
• **Scenario 2**: nonzero coefficients of $B_{\left(1\right)}$ and $B_{\left(2\right)}$ are **-0.5**, nonzero coefficients of $B_{\left(t\right)}$ (t=3,⋯,T) are **0.5.**
• **Scenario 3**: nonzero coefficients of $B_{\left(1:2\right)}$ are **0.4**, $B_{\left(3:4\right)}$ are **0.6**, $B_{\left(5:6\right)}$ are **0.8**, $B_{\left(7:8\right)}$ are **1.0** and $B_{\left(9:10\right)}$ are **1.2**, where $B_{\left(a:b\right)}$ represents both $B_{\left(a\right)}$ and $B_{\left(b\right)}$.
• **Scenario 4**: nonzero coefficients of $B_{\left(1\right)}$ are **-0.7**, $B_{\left(2\right)}$ are **-0.5**, $B_{\left(3\right)}$ are **-0.3**, $B_{\left(4\right)}$ are **0.2**, $B_{\left(5\right)}$ are **0.4**, $B_{\left(6\right)}$ are **0.6**, $B_{\left(7\right)}$ are **0.8**, $B_{\left(8\right)}$ are **1.0**, $B_{\left(9\right)}$ are **1.2** and $B_{\left(10\right)}$ are **1.4.**

After training the models in the simulated data, we additionally simulated n=300 samples for validation of the prediction accuracy. As an evaluation metric, we calculated Spearman’s ρ between each sample group (e.g., cancer tissue type) and each response variable (e.g., drug) to investigate the rank correlation between the observed responses and the model-predicted responses in the validation set. We ran 50 simulations, and for each sample group and each response variable (i.e. drug response) the Spearman’s ρ was averaged over the 50 simulations. We also used another evaluation metric, Root Mean Squared Error (RMSE), for evaluating the accuracy of predicting continuous drug response levels, in addition to the ranking accuracy as evaluated by the Spearman’s ρ.

In scenario 1, where the multiple sample groups share the same covariate effects, tree lasso and mix-lasso have similar prediction accuracy (Wilcoxon test p=0.071; Figure S2.1a). In a more challenging scenario 2, where the first two sample groups have opposite covariate effects (i.e. negative and positive regression coefficients) compared to the other groups, mix-lasso shows much better prediction accuracy compared to tree lasso (p<0.001). In scenario 3, where the covariate effects are different across the sample groups, mix-lasso has again similar prediction performance to that of tree lasso (p=0.533). In scenario 4, where the heterogeneous sample groups have both positive and negative effects and varying scales, mix-lasso shows again much better prediction accuracy compared to tree lasso (p<0.001). RMSE shows similar conclusions than Spearman’s ρ in scenarios 2 and 4, while tree lasso outperforms mix-lasso in scenarios 1 and 3 based on RMSE (see Figure S2.2). These results indicate that mix-lasso results in better prediction performance than tree lasso in cases, where there exist heterogeneous feature effects in different sample groups, especially when there are opposite effects of the same features in different sample groups.

To evaluate the feature selection performance of the two models, we used a receiver operating characteristic (ROC) curve to investigate if the estimated coefficient of a covariate is truly nonzero or zero, compared to the ground-truth simulation model. Figure S2.1 shows that mix-lasso and tree lasso have very similar feature selection accuracy w.r.t. the area under the receiver operating characteristic curve (AUC) in scenarios 1, 3 and 4. However, similar to the prediction accuracy, mix-lasso shows a much better AUC value than tree lasso in the more challenging scenario 2, where there exist opposite effects of the same features in different sample groups. This indicates that the mix-lasso accurately identifies relevant features for drug responses across multiple tissue types, especially when there is strong heterogeneity between sample groups, e.g., if the same feature may have opposite effects in two patient groups of cancer types.

## Data Availability

• Data: The CTRP v2 pharmacological data are publicly available at https://ocg.cancer.gov/programs/ctd2/data-portal. The corresponding genomic data were obtained using the freely available R package PharmacoGx (Smirnov et al., 2016). • Code: The R code for the CTRP and simulated data analysis have been made available at https://github.com/zhizuio/mixlasso_example. The R-package **mixlasso** for our mix-lasso model is available on GitHub at https://github.com/zhizuio/mixlasso. • Any additional information required to reanalyze the data reported in this paper is available from the lead contact upon request.
