# Supplementary material for: Tissue-specific identification of multi-omics features for pan-cancer drug response prediction
Source: iScience. 2022 Jul 19;25(8):104767. doi: 10.1016/j.isci.2022.104767 (PMC9385562; doi:10.1016/j.isci.2022.104767)
Supplement: Document S1. Figures S1 and S2 and Table S1 [file mmc1.pdf]

iScience, Volume *25*

## Supplemental information

# Tissue-specific identification of multi-omics features for pan-cancer drug response prediction

**Zhi Zhao, Shixiong Wang, Manuela Zucknick, and Tero Aittokallio**

# S1: Additional results of the CTRP data analysis

Table S1 *Overall prediction performance (mean + standard deviation) with various CTRP omics data*

| | mix-lasso | | | tree lasso | | |
|---|---|---|---|---|---|---|
| Performance | GEX+CNV+MUT | GEX+MUT | $P$-value | GEX+CNV+MUT | GEX+MUT | $P$-value |
| RMSE | 1.640 (0.072) | 1.517 (0.117) | 0.028 | 0.945 (0.042) | 0.947 (0.045) | 0.739 |
| Pearson's $r$ | 0.025 (0.201) | 0.023 (0.159) | 0.971 | 0.039 (0.149) | 0.038 (0.136) | 0.912 |
| Spearman's $\rho$ | 0.013 (0.194) | 0.026 (0.180) | 0.912 | 0.024 (0.128) | 0.025 (0.114) | 0.971 |

NOTE: All values were calculated by averaging w.r.t. individual cancer tissue types and individual drugs based 10 split validation data. Small RMSE values indicate better prediction accuracy, whereas large Pearson's $r$ and Spearman's $\rho$ correlations indicate improved accuracy. Wilcoxon rank sum test was used to compare each evaluation metric when using either three omics data or two omic data.

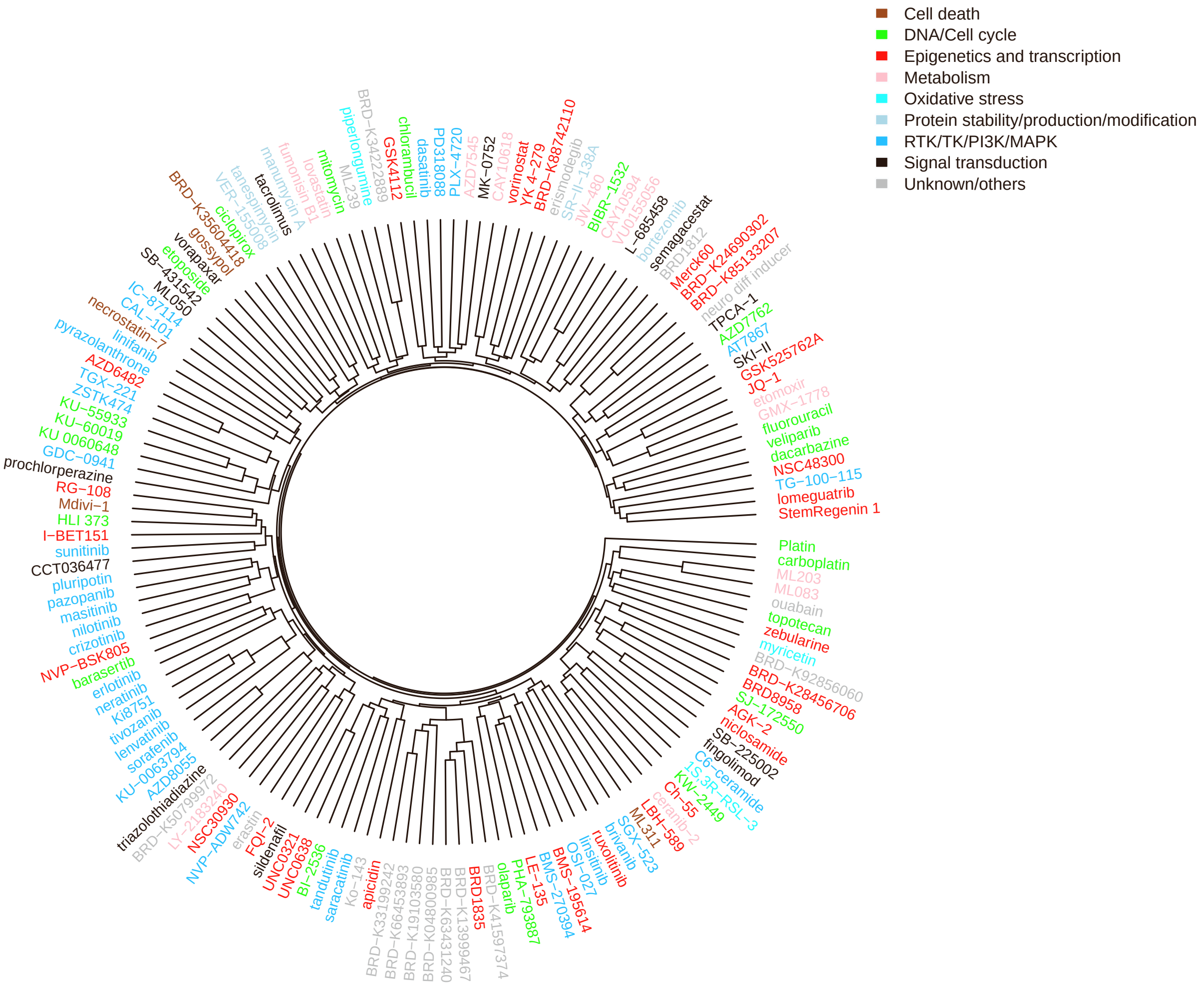


Figure S1.1: Hierarchical clustering of the CTRP drug responses was carried out using hierarchical agglomerative complete-linkage clustering with Tanimoto index as similarity measure over the drug SMILES fingerprints.

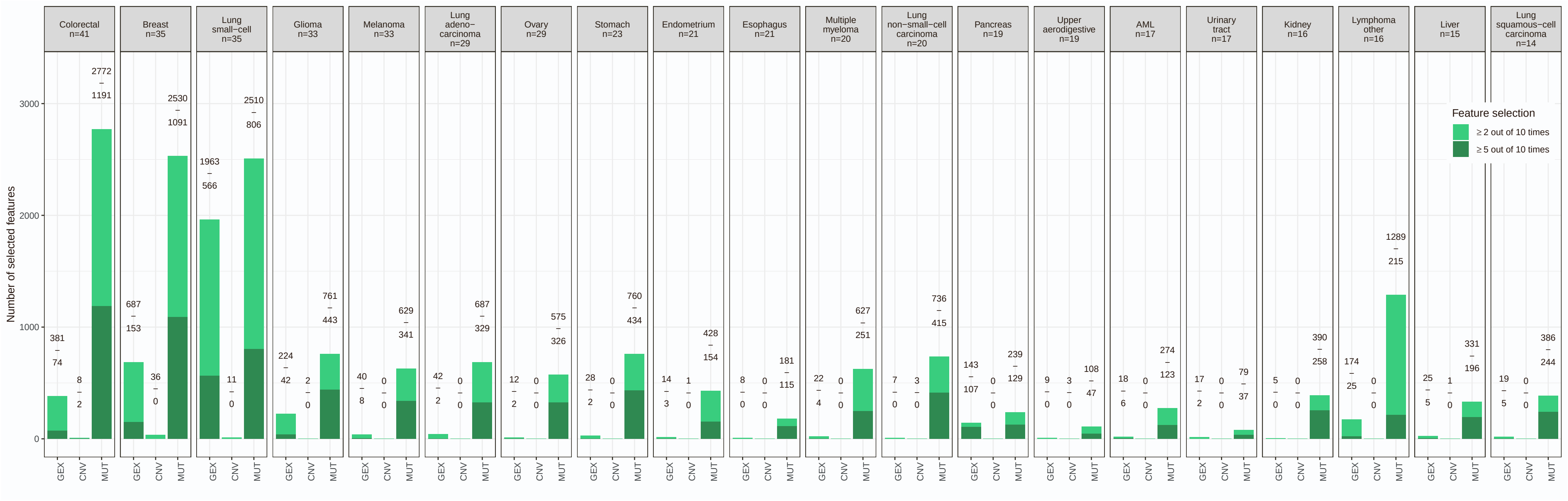


Figure S1.2: Feature selection by mix-lasso over the $m_{\text{drugs}} = 147$ common drugs with respect to the three omics data sources and 20 cancer types. The total numbers of features for individual data sources were $m_{\text{drugs}} \times p_{\text{GEX}} \approx 3.0 \times 10^5$, $m_{\text{drugs}} \times p_{\text{CNV}} \approx 1.2 \times 10^6$, and $m_{\text{drugs}} \times p_{\text{MUT}} \approx 2.6 \times 10^4$. The green (dark green) bar shows the numbers of features selected when one of the model coefficients was nonzero at least 2 (5) out of 10 times. The cancer types are ordered by the sample sizes from colorectal ($n = 41$) to lung squamous cell carcinoma ($n = 14$).

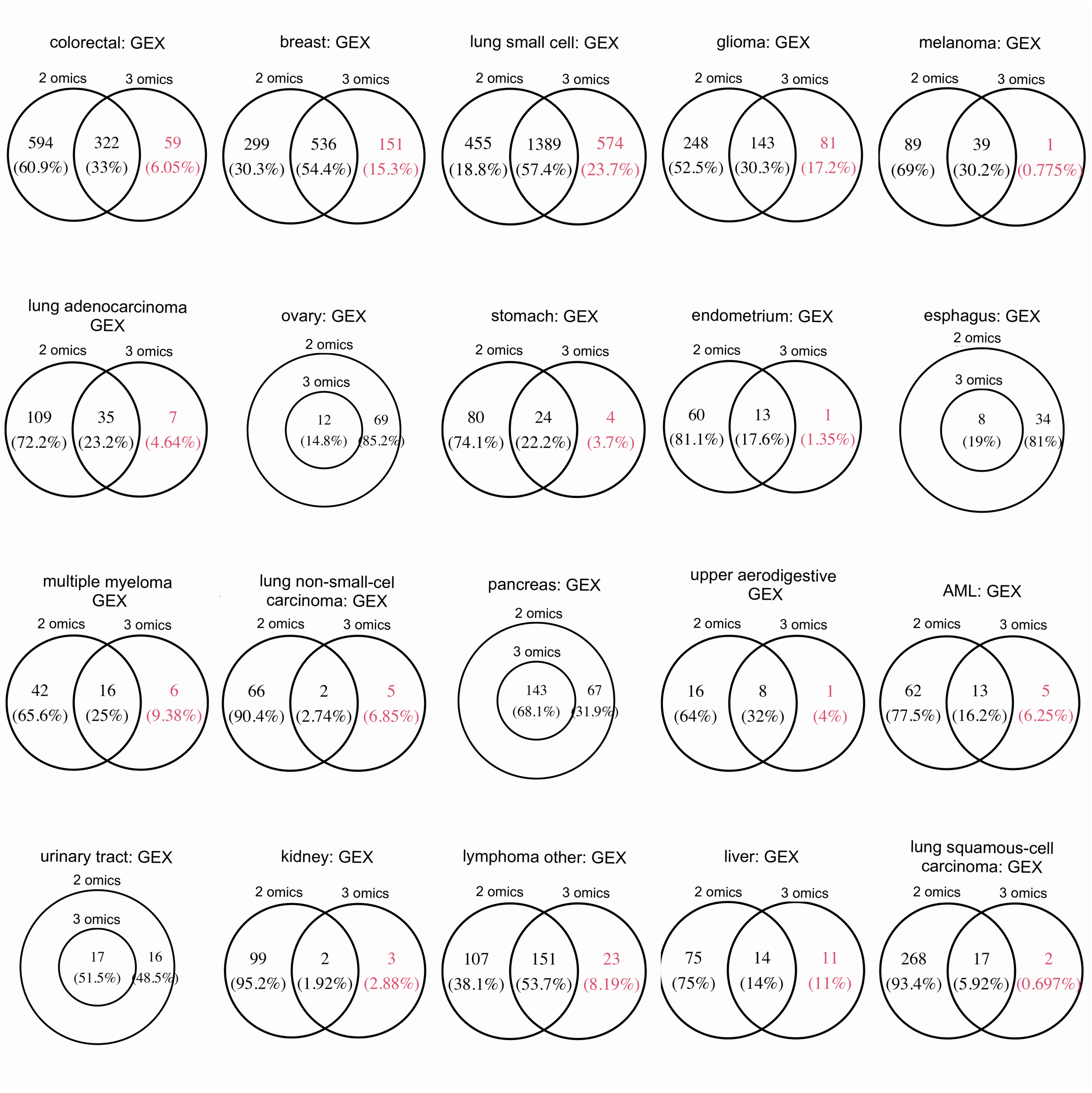


Figure S1.3: Overlapping GEX features selected when modelling three omics data and when modelling two omics data. Red numbers or percentages show the GEX features selected when modelling three omics data but not selected when modelling two omics data, which are relative small.

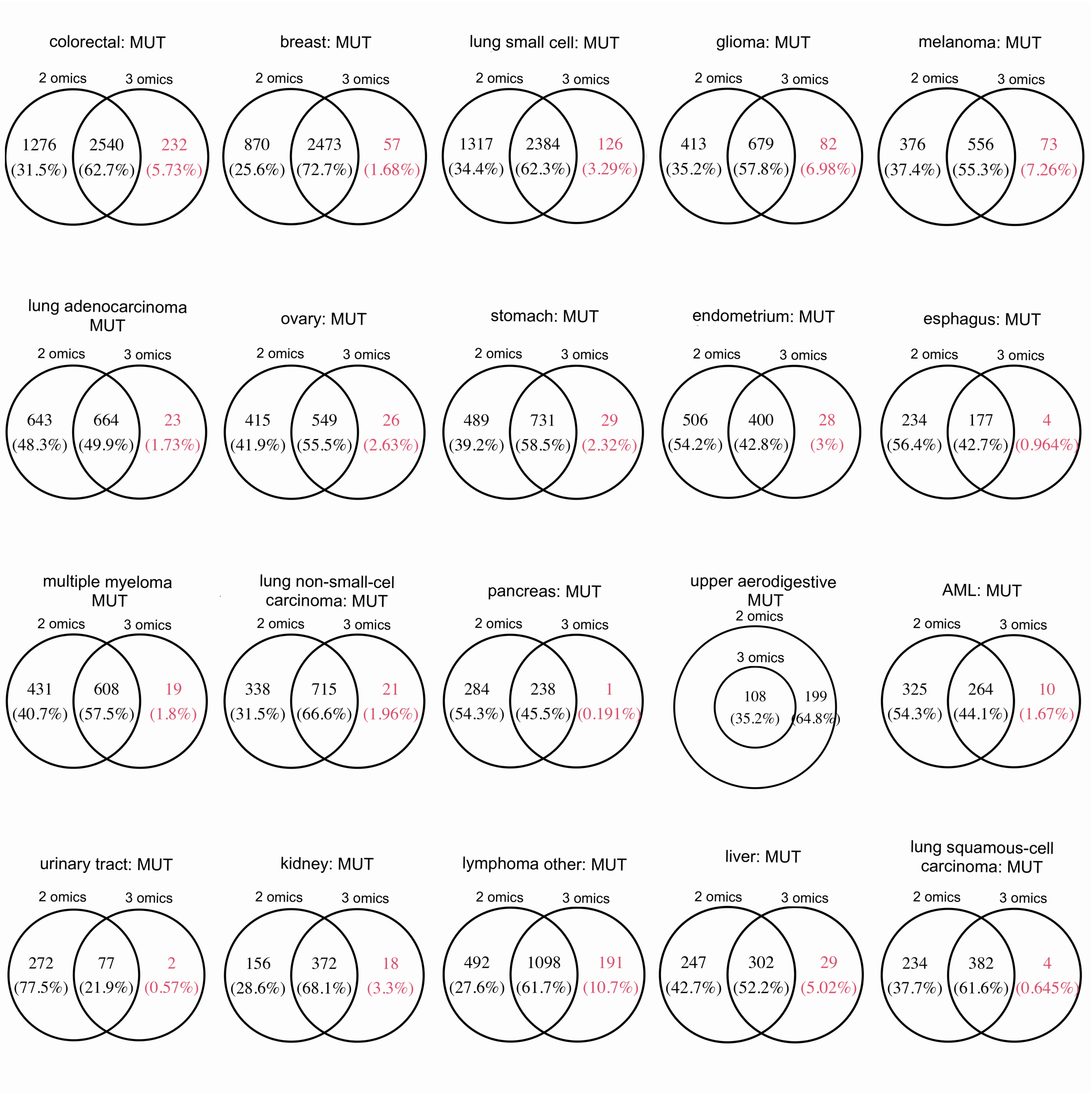


Figure S1.4: Overlapping MUT features selected when modelling three omics data and when modelling two omics data. Red numbers or percentages show the MUT features selected when modelling three omics data but not selected when modelling two omics data, which are relative small.

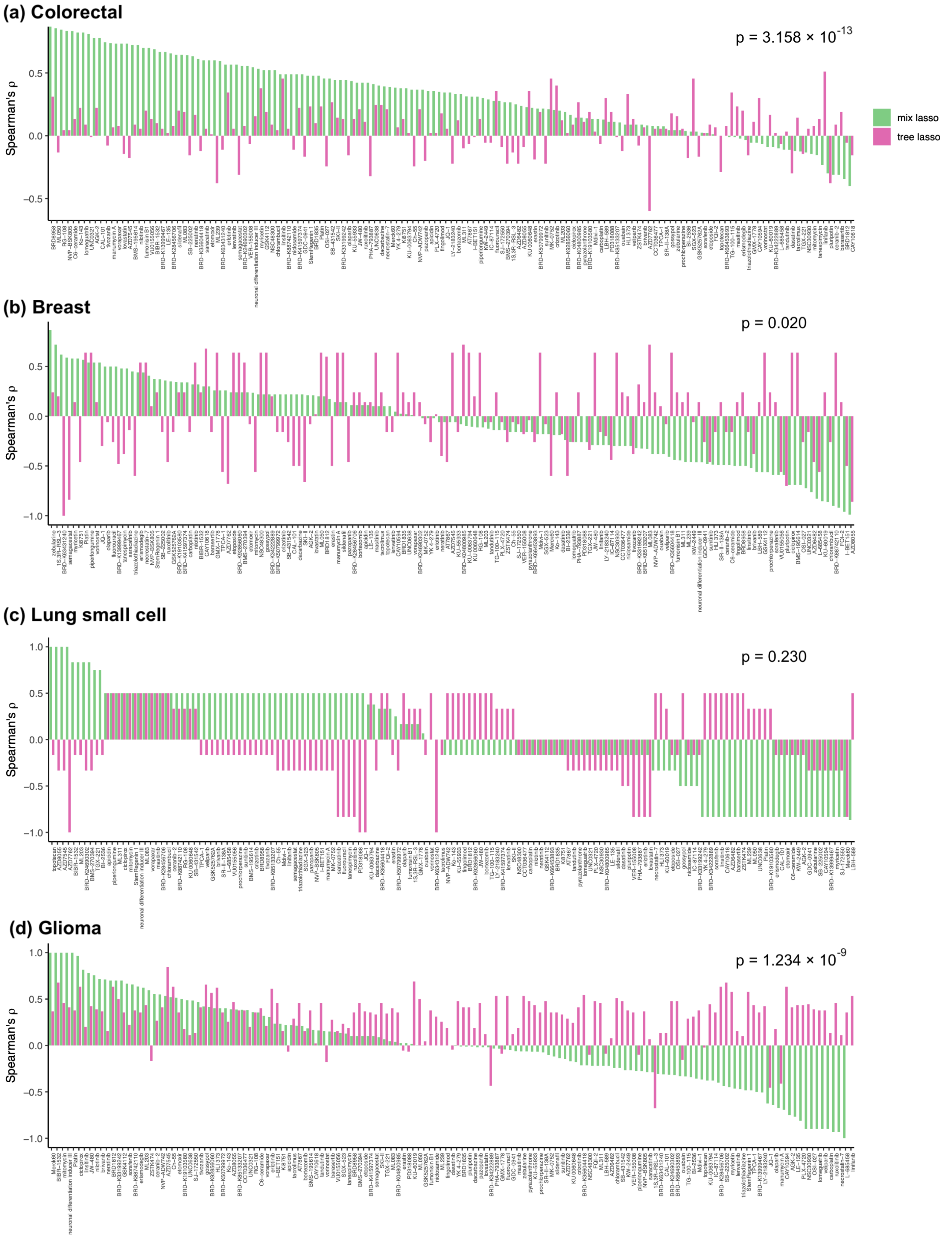

(a) Colorectal
p = 3.158 × 10^-13
Spearman's ρ
mix lasso
tree lasso
(b) Breast
p = 0.020
Spearman's ρ
(c) Lung small cell
p = 0.230
Spearman's ρ
(d) Glioma
p = 1.234 × 10^-9
Spearman's ρ

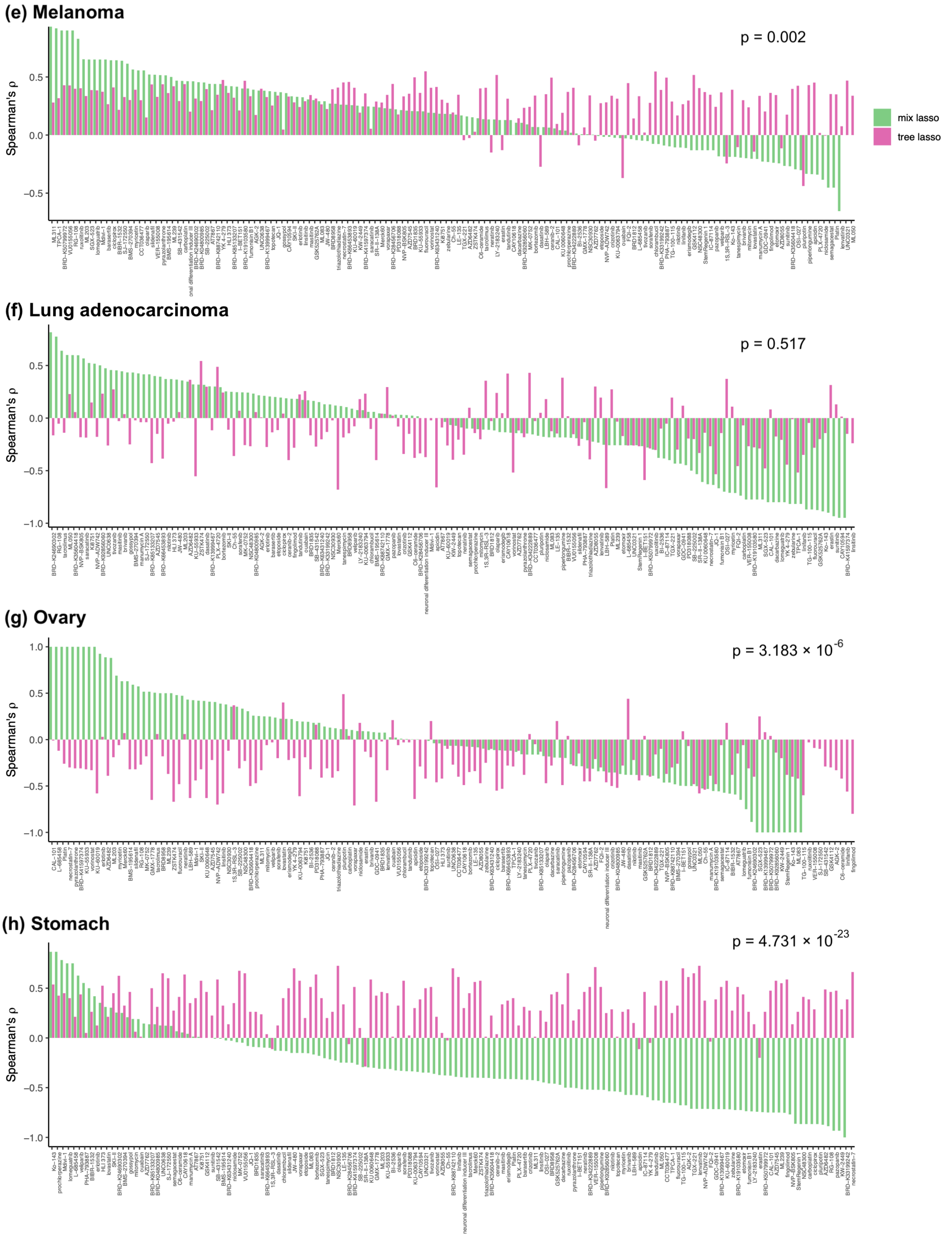

(e) Melanoma
p = 0.002
mix lasso
tree lasso
Spearman's ρ
(f) Lung adenocarcinoma
p = 0.517
Spearman's ρ
(g) Ovary
p = 3.183 × 10^-6
Spearman's ρ
(h) Stomach
p = 4.731 × 10^-23
Spearman's ρ

### (i) Endometrium

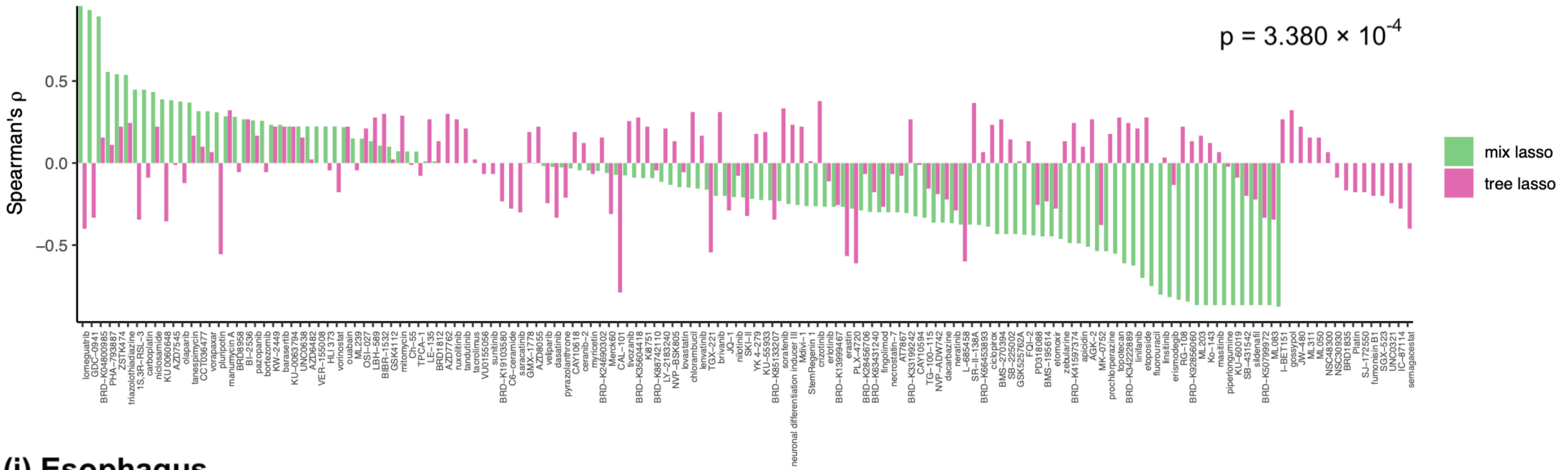


### (j) Esophagus

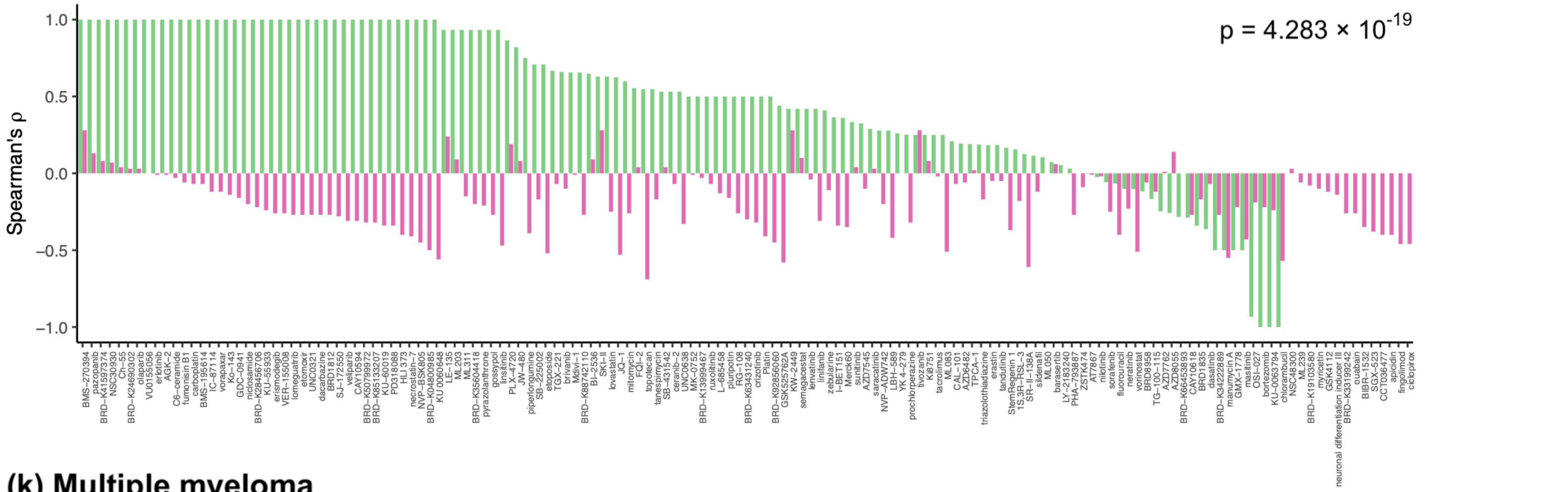


### (k) Multiple myeloma

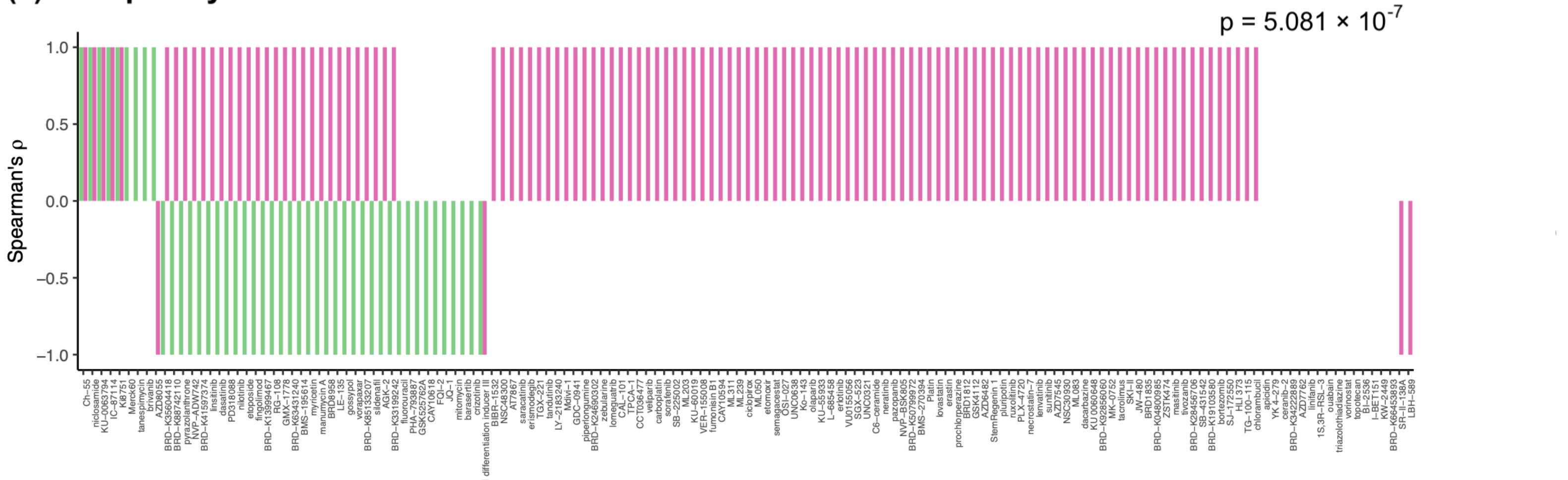


### (l) Lung non-small-cell carcinoma

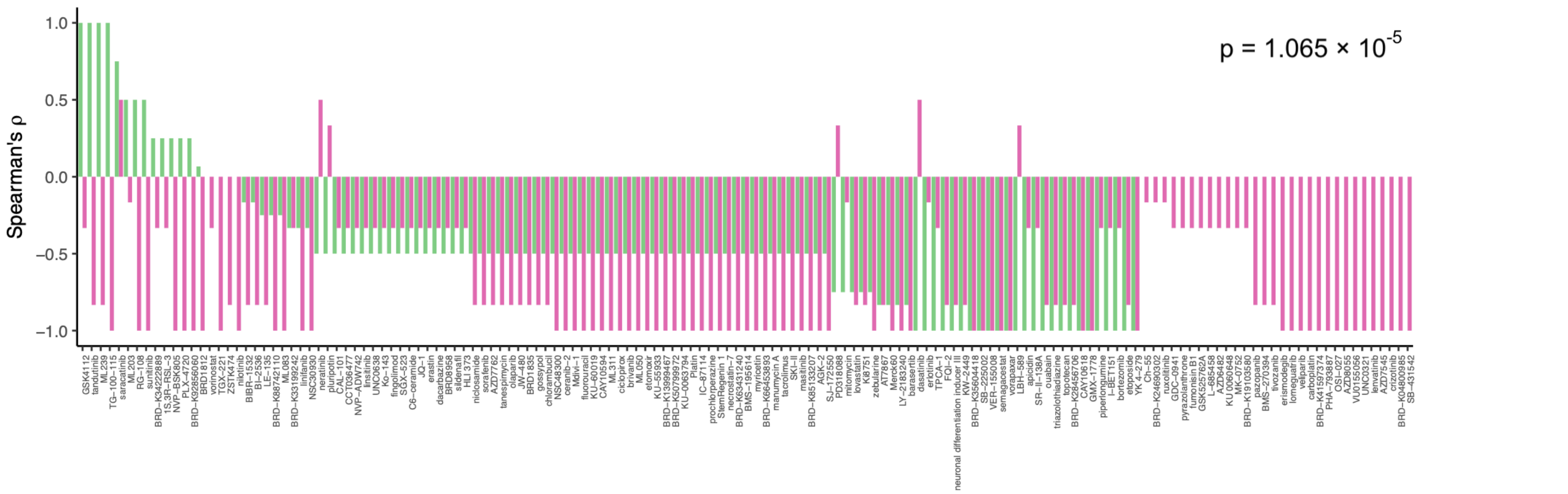

## (m) Pancreas

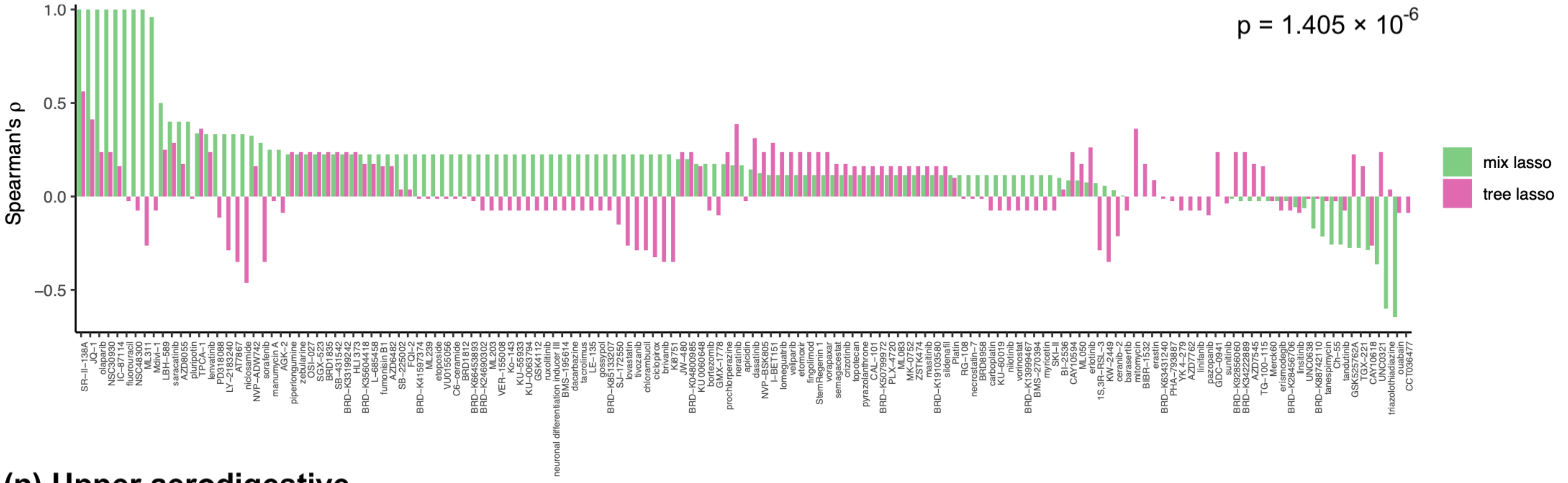


## (n) Upper aerodigestive

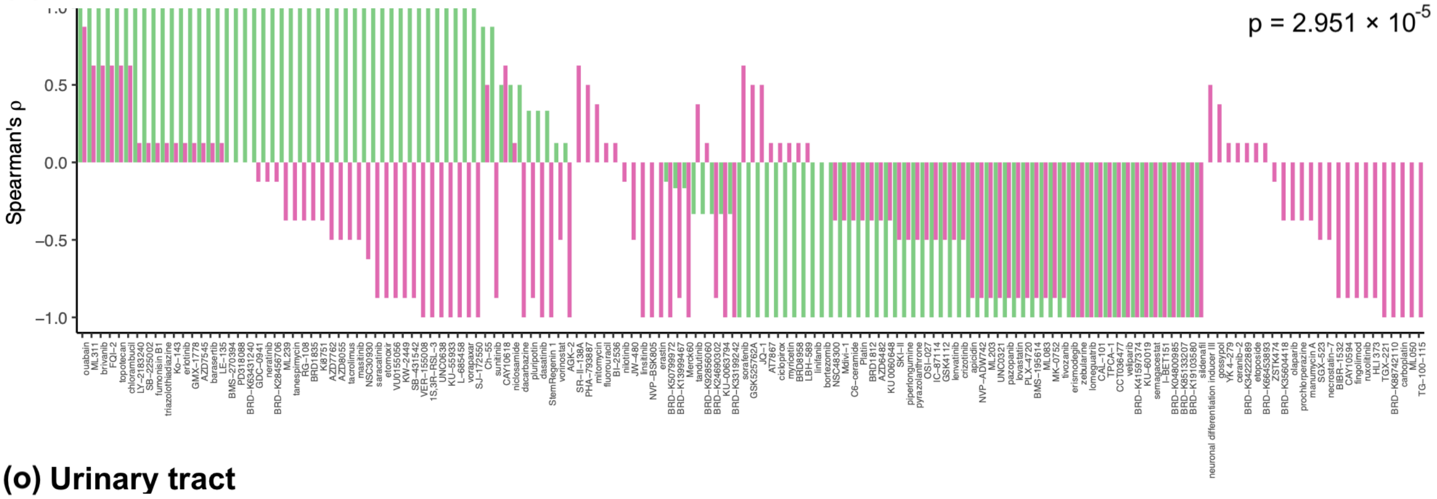


## (o) Urinary tract

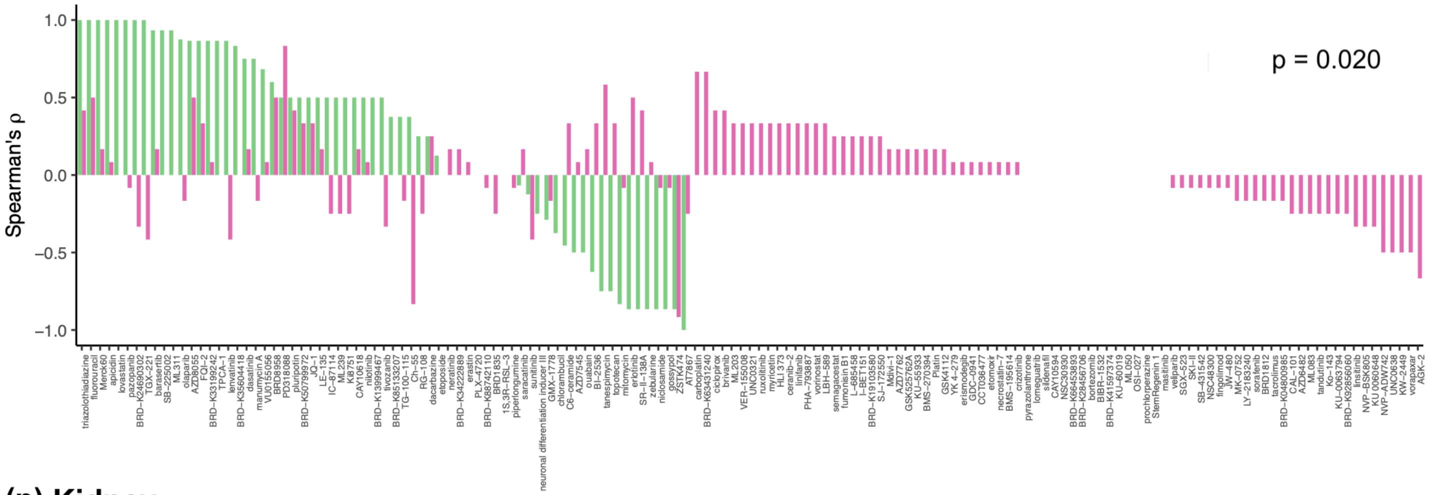


## (p) Kidney

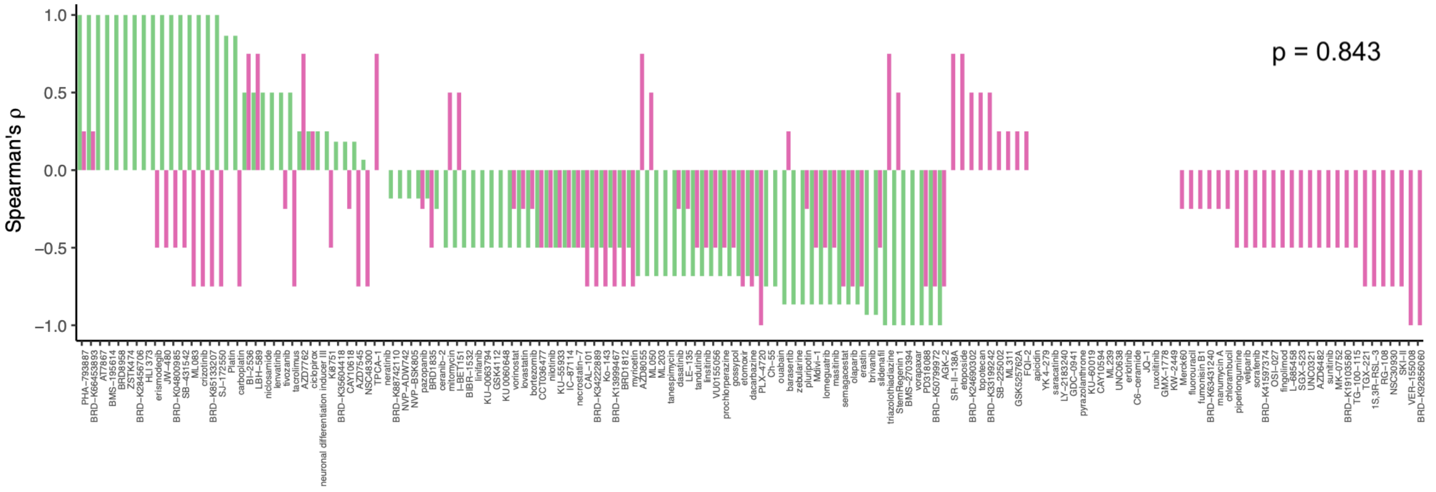

**(q) Lymphoma other**

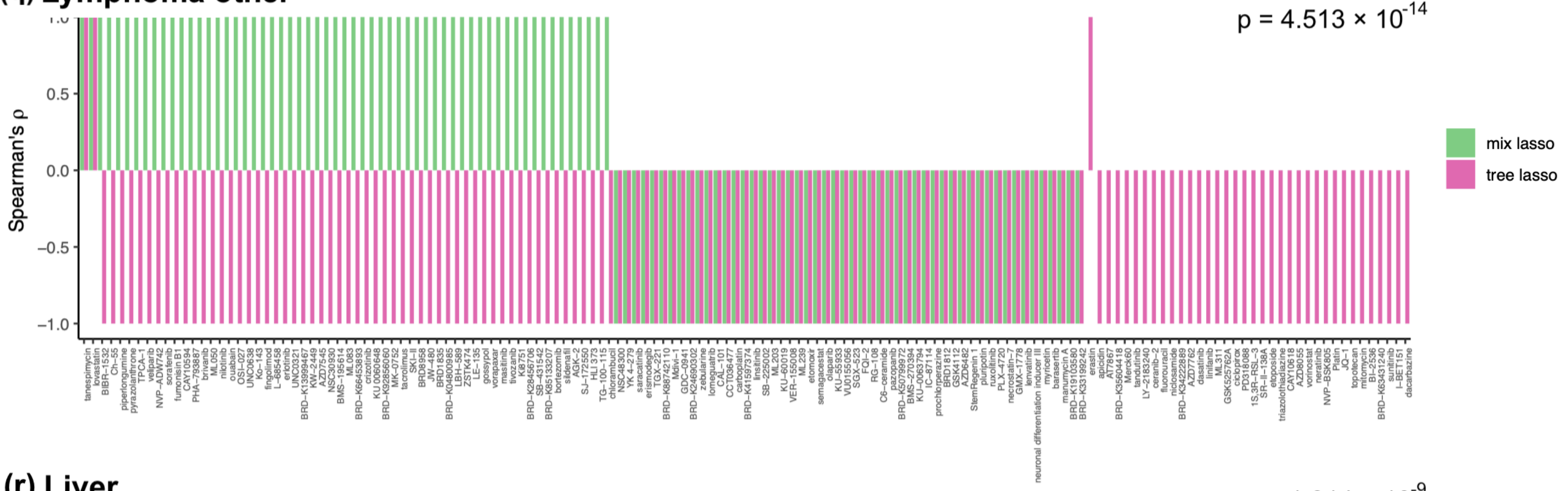


**(r) Liver**

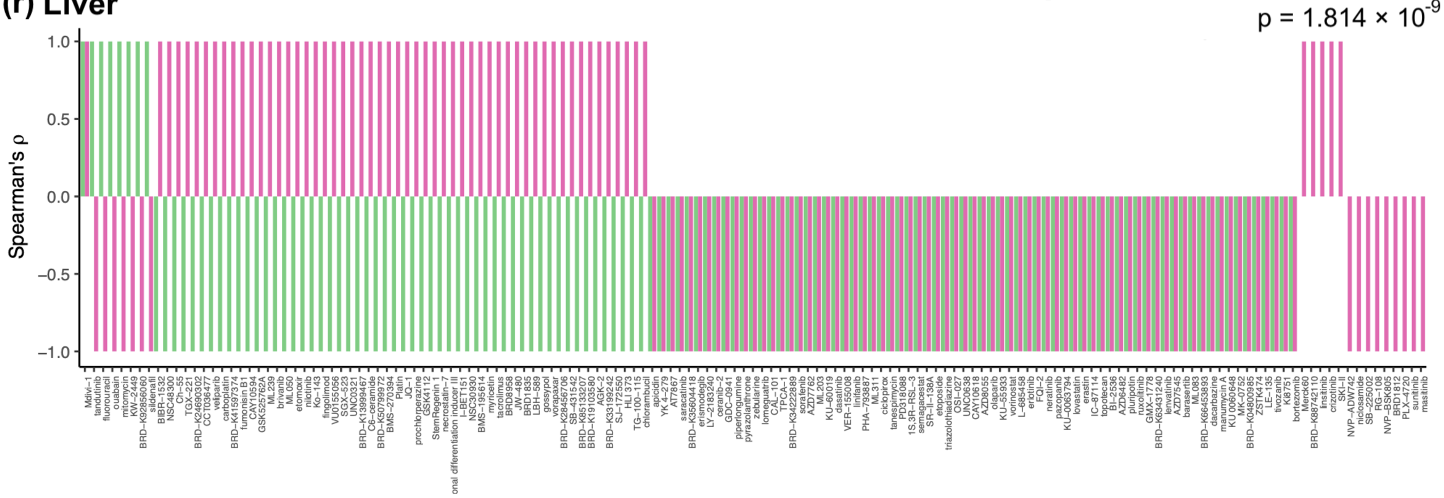


**(s) Lung squamous-cell carcinoma**

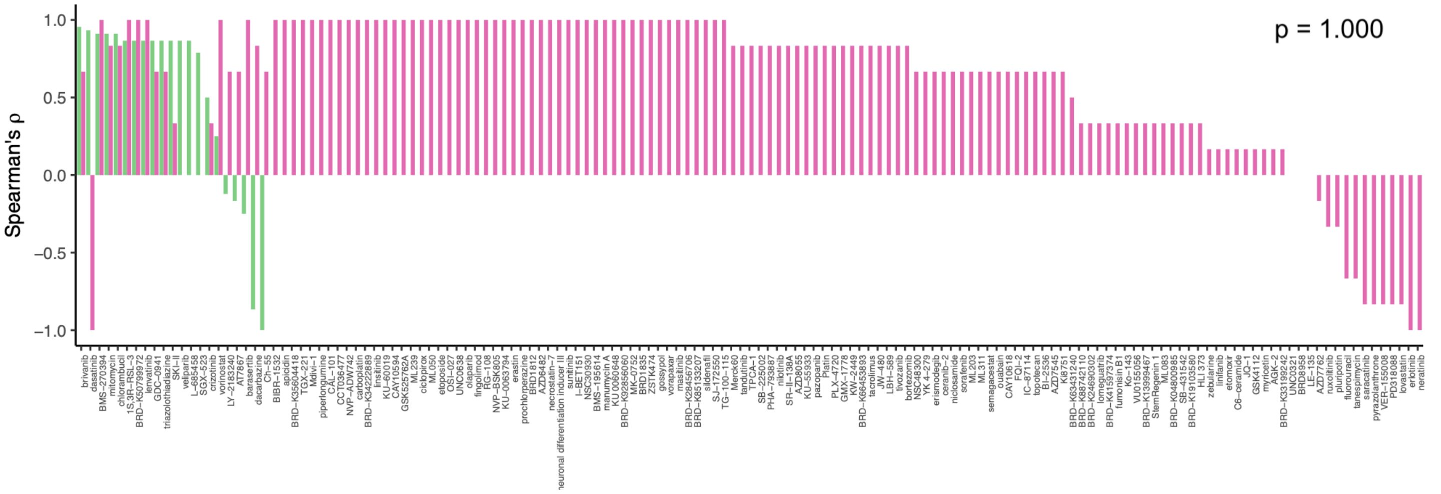


Figure S1.5: Response prediction accuracy (Spearman's $\rho$) for cell lines of the 19 cancers (a)-(s), i.e. colorectal, breast, lung small cell, glioma, melanoma,lung adenocarcinoma, ovary, stomach, endometrium, esophagus,multiple myeloma, lung non-small-cell carcinoma, pancreas, upper aerodigestive, urinary tract, kidney, lymphoma other, liver and lung squamous cell carcinoma cancers, w.r.t. the 147 drugs with mix-lasso and tree lasso for joint analysis of all cancer types. No AML cancer type results are shown because there are not enough AML cell lines for prediction in validation data. $P$-value compare difference between mix-lasso and tree lasso prediction accuracy calculated based on the Wilcoxon signed ranks test w.r.t. cancer types.

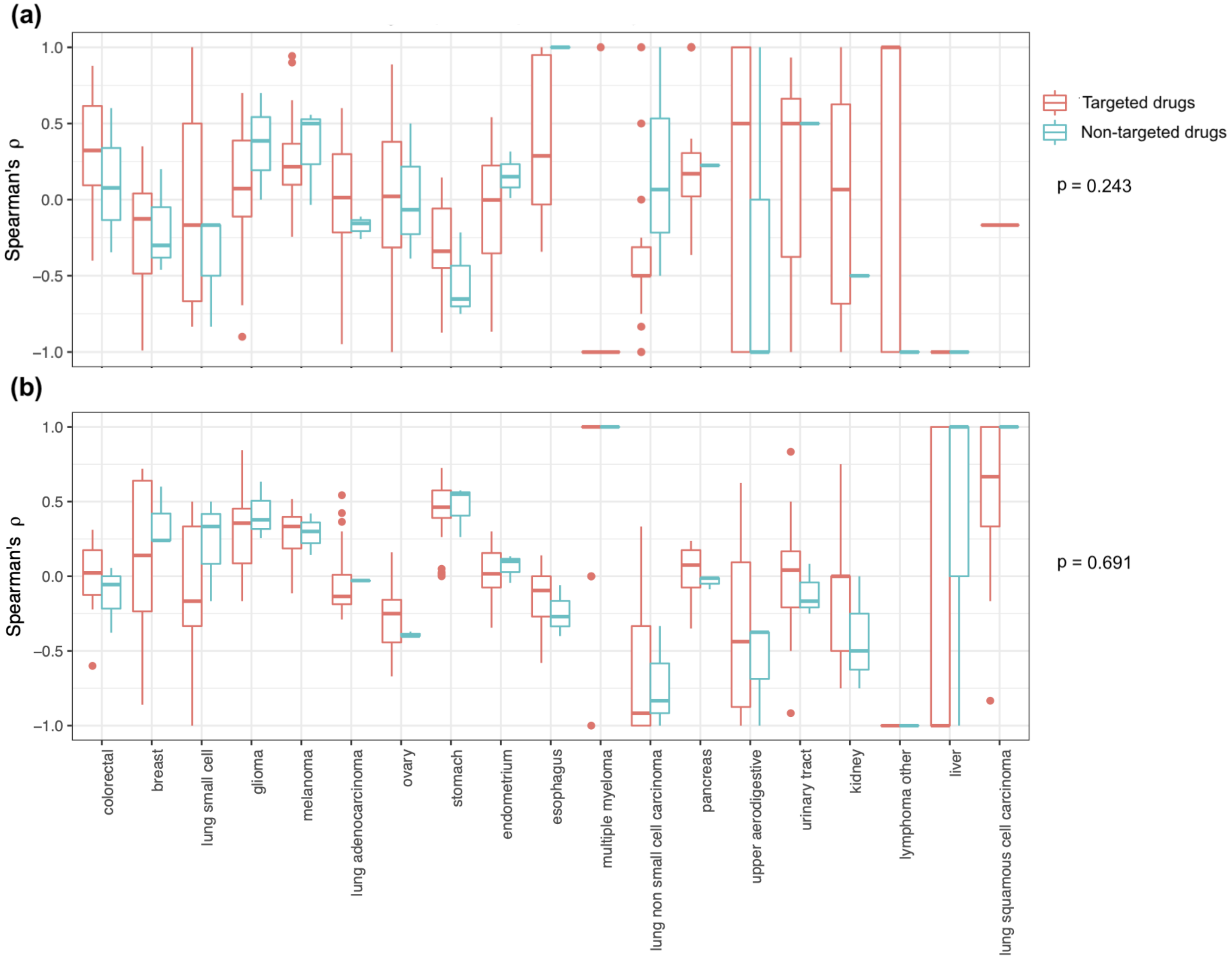


Figure S1.6 Response prediction accuracy (Spearman's $\rho$) for targeted and non-targeted drugs w.r.t. the 19 cancer types by (a) mix-lasso and (b) tree lasso. Note that the non-targeted drug responses of the AML are missing due to limited number of cell lines in the validation data. Wilcoxon signed rank test was used to compare the difference of Spearman's $\rho$ between targeted and non-targeted drugs.

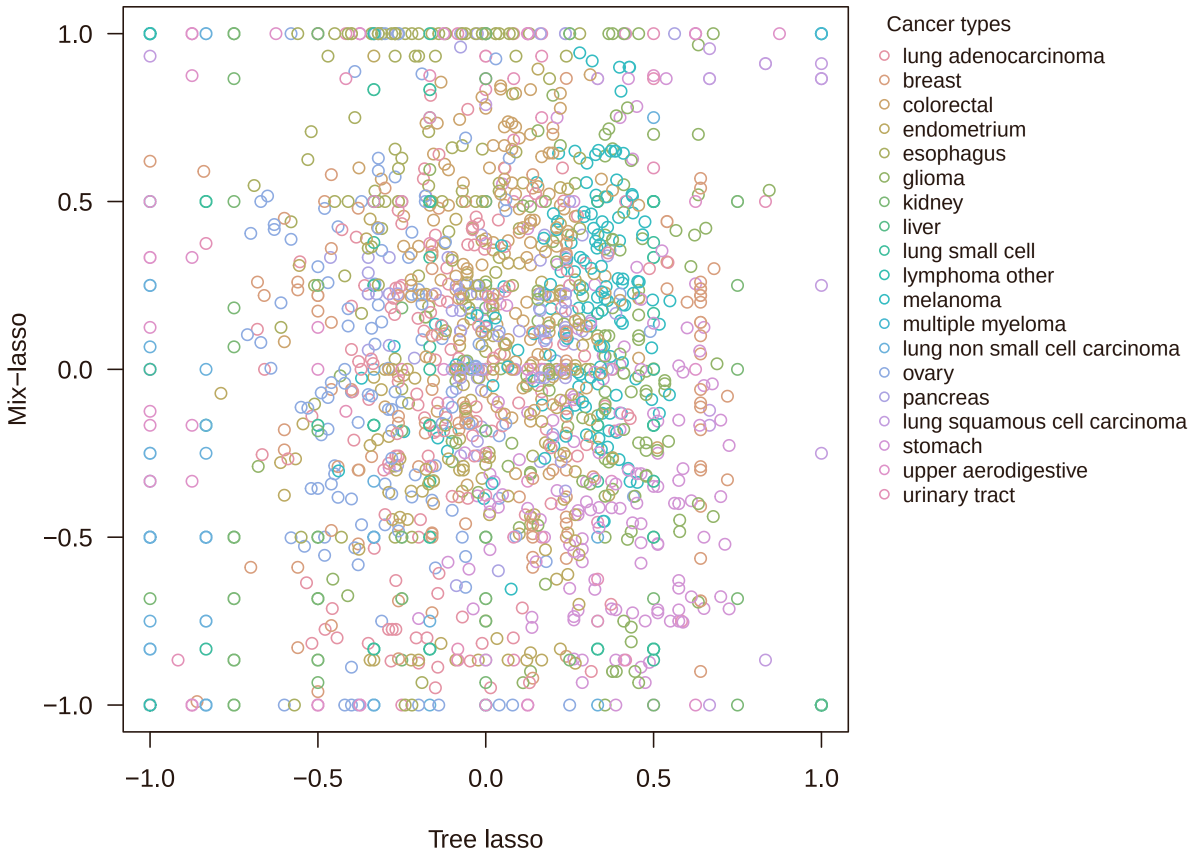


Figure S1.7 Comparison of the prediction accuracy (Spearman correlation) between mix-lasso and tree lasso across the 19 cancer types. No AML cancer type results are shown because there are not enough AML cell lines for prediction in the validation data.

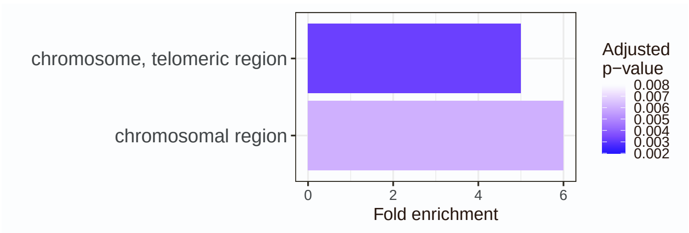


Figure S1.8 Enrichment of GO cellular components based on the selected genes corresponding to JQ-1 for colorectal and melanoma cancers. $P$-values were adjusted for multiple testing by controlling the false discovery rate with the Benjamini & Hochberg method.

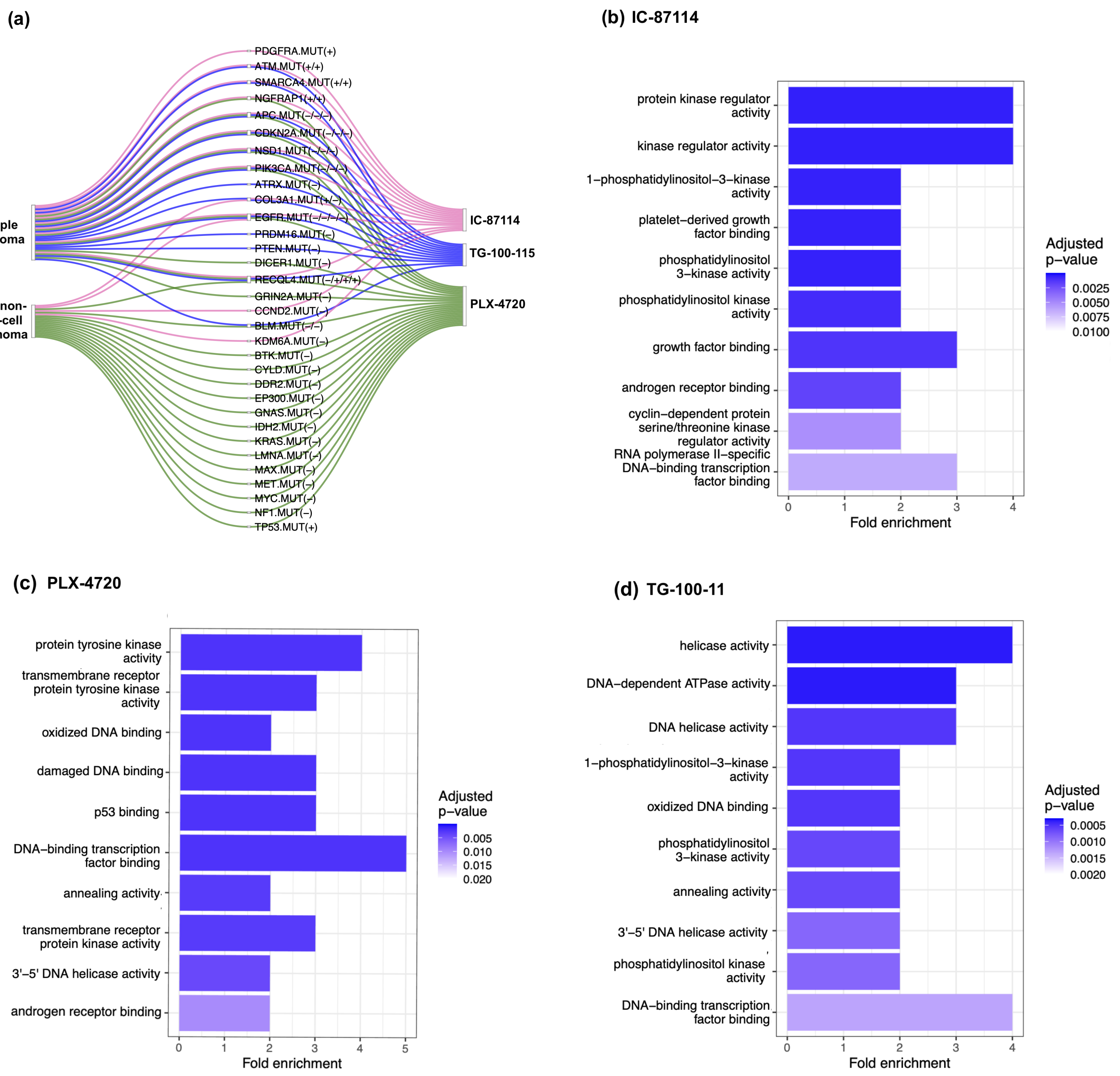


Figure S1.9 (a) Example drugs IC-87114, PLX-4720 and TG-100-115, and their identified genomic (MUT) and molecular features (GEX) linked to the three selected cancer types by mix-lasso. The selected genes were based on feature selection criteria "$\geq$ 2 out of 10 times". Gene names with ".MUT" indicate MUT features and the rest are GEX features. "+" or "-" indicates positive or negative effect. The multiple signs correspond to distinct gene-cancer-drug response relationships (i.e., the number of connections in the sankey diagram). Note that the negative effect of "COL3A1.MUT" corresponds to drug PLX-4720 on multiple myeloma cancer. (b) Enrichment of GO molecular functions among the mix-lasso-selected genes predictive of responses to drug IC-87114 and (c) to drug PLX-4720 and (d) to drug TG-100-115. $P$-values in panels (b-d) were adjusted for multiple testing by controlling the false discovery rate with the Benjamini & Hochberg method.

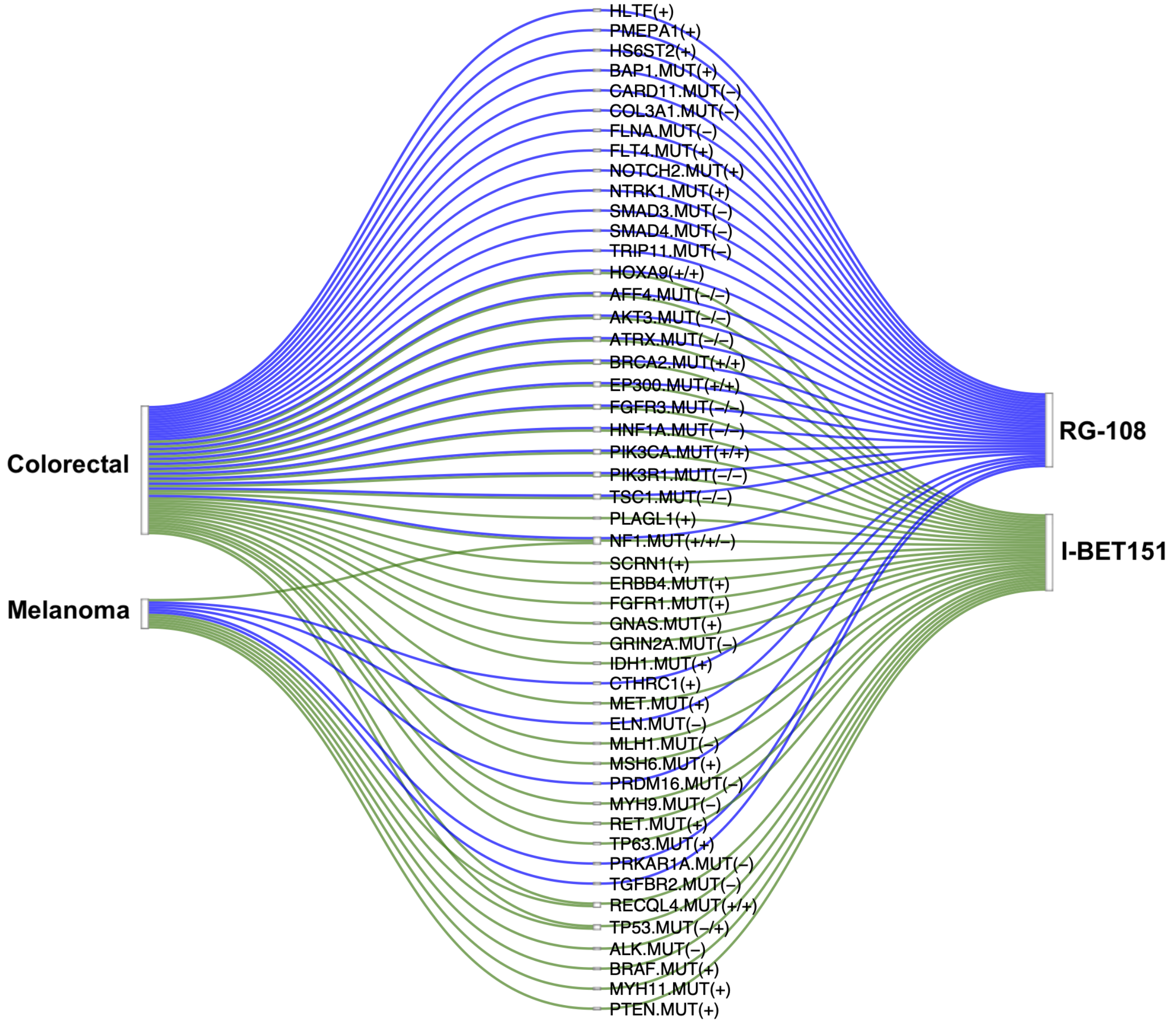


Figure S1.10 Example drugs RG-108 and I-BET151 and their identified genomic (MUT) and molecular features (GEX) linked to the two selected cancer types by mix-lasso. The selected genes were based on feature selection criteria "$\geq$ 2 out of 10 times". Gene names with ".MUT" indicate MUT features and the rest are GEX features. "+" or "-" indicates positive or negative effect. The multiple signs correspond to distinct gene-cancer-drug response relationships (i.e., the number of connections in the sankey diagram). Note that the negative effect of "NF1.MUT" corresponds to drug I-BET151 in melonoma, and the negative effect of "TP53.MUT" corresponds to drug I-BET151 in colorectal cancer.

# S2: Simulation results

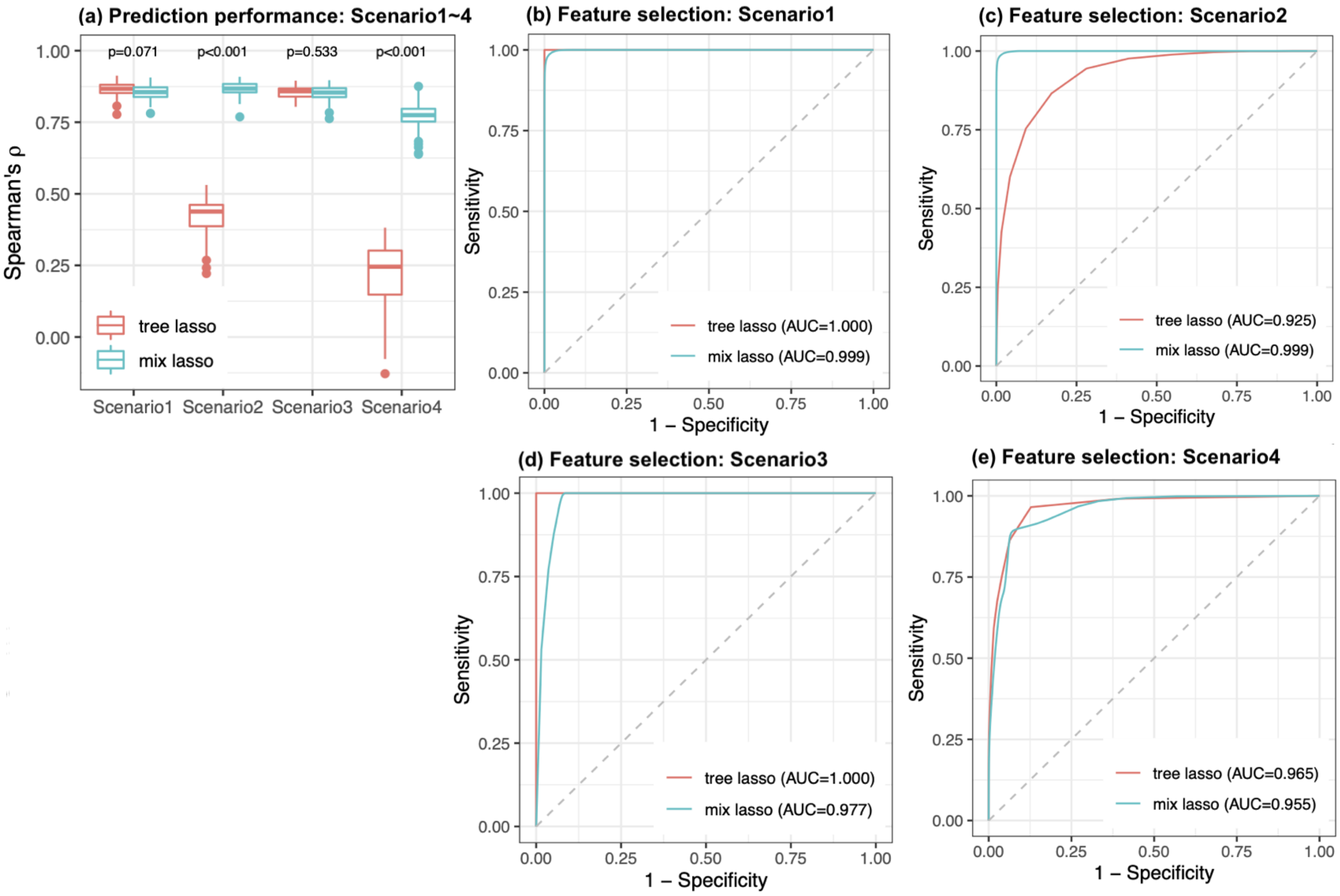


Figure S2.1 Comparison of prediction performance and feature selection of tree lasso and mix-lasso. (a) Each point in the box plot represents an averaged Spearman's $\rho$ over the 50 simulations w.r.t. each sample group $t = 1, \cdots, T$ and each response variable (i.e. drug response) $k = 1, \cdots, m$. Statistical comparison of the Spearman's $\rho$ was done by Wilcoxon test. (b-d) Sensitivity and specificity of feature selection were calculated by measuring the recovery of true zero and nonzero elements in the coefficients matrix, respectively. The area under the receiver operating characteristic curve (AUC) is shown in parentheses. The gray dashed line represents the performance of a random classifier with AUC = 0.5.

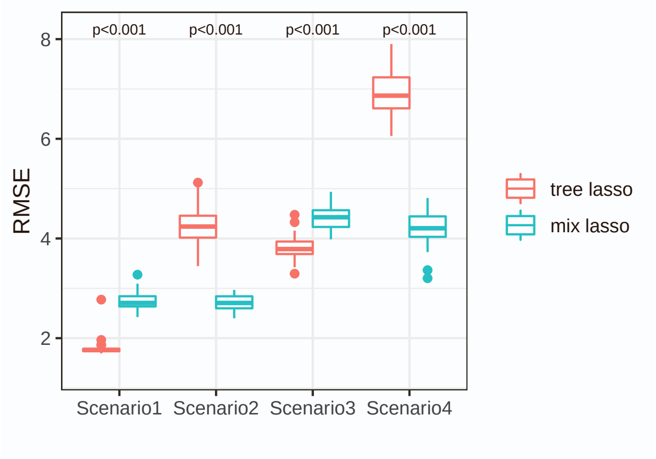


Figure S2.2 Comparison of prediction performance w.r.t. RMSE based on 50 simulated validation data, with $n = 300$, $m = 120$, $T = 10$ and $p = 1000$.
